# Assessment of a reconfiguration of the InterSpread Plus US national FMD model as a potential tool to analyze a foot-and-mouth disease outbreak on a single large cattle feedlot in the United States

**DOI:** 10.3389/fvets.2023.1205485

**Published:** 2023-08-16

**Authors:** Sarah R. Mielke, Columb Rigney, Amy D. Hagerman, Timothy C. Boyer, Amy H. Delgado, Jonathan Arzt, Lindsey K. Holmstrom

**Affiliations:** ^1^United States Department of Agriculture’s Animal and Plant Health Inspection Service (APHIS), Fort Collins, CO, United States; ^2^Department of Agricultural Economics, Oklahoma State University, Stillwater, OK, United States; ^3^Foreign Animal Disease Research Unit, USDA-ARS, Plum Island Animal Disease Center, Greenport, NY, United States; ^4^United States Department of Agriculture’s Animal and Plant Health Inspection Service (APHIS), Riverdale, MD, United States

**Keywords:** FMD (foot-and-mouth disease), disease response strategies, cattle feedlot, United States, modeling

## Abstract

**Introduction:**

An incursion of foot-and-mouth disease (FMD) into the United States remains a concern of high importance and would have devastating socioeconomic impacts to the livestock and associated industries. This highly transmissible and infectious disease poses continual risk for introduction into the United States (US), due to the legal and illegal global movement of people, animals, and animal products. While stamping out has been shown to effectively control FMD, depopulation of large cattle feedlots (>50,000 head) presents a number of challenges for responders due to the resources required to depopulate and dispose of large numbers of animals in a timely and effective manner.

**Methods:**

However, evaluating alternative strategies for FMD control on large feedlots requires a detailed within-farm modeling approach, which can account for the unique structure of these operations. To address this, we developed a single feedlot, within-farm spread model using a novel configuration within the InterSpread Plus (ISP) framework. As proof of concept we designed six scenarios: (i) *depopulation* - the complete depopulation of the feedlot, (ii) *burn-through* – a managed “burn-through” where the virus is allowed to spread through the feedlot and only movement restriction and biosecurity are implemented, (iii) *firebreak-NV* – targeted depopulation of infected pens and adjacent pens without vaccination; (iv) *firebreak* - targeted depopulation of infected pens and adjacent pens with vaccination of remaining pens; (v) *harvest-NV* - selective harvest of pens where a 100% movement restriction is applied for 28-30 days, then pens are set for selection to be sent to slaughter, while allowing a controlled “burn-through” without vaccination; and (vi) *harvest* - selective harvest of pens with vaccination.

**Results:**

Overall, the burn-through scenario (ii) had the shortest epidemic duration (31d (30, 33)) median (25th, 75th percentiles), while the firebreak scenario (iv) had the longest (47d (38,55)). Additionally, we found that scenarios implementing depopulation delayed the peak day of infection and reduced the total number of pens infected compared to non-depopulation scenarios.

**Discussion:**

This novel configuration of ISP provides proof of concept for further development of this new tool to enhance response planning for an incursion of FMD in the US and provides the capability to investigate response strategies that are designed to address specific outbreak response objectives.

## 1. Introduction

The response strategy to a foot-and-mouth disease outbreak in the United States (US) focuses on detection, control, and containment (1). The goal is to eradicate the disease with minimal instability felt throughout animal agriculture, the food supply chain, and economy using science and risk-based strategies, while protecting public health and the environment (1). Among these strategies is stamping out, a response strategy routinely used to stabilize the industry by minimizing spread of this highly contagious pathogen. However, response strategies continue to be evaluated as science and knowledge advance and allow us to address complexities in different livestock production types. An area of interest is the feasibility of culling and disposal on large feedlots, which can house more than 50,000 head of cattle.

In the US agricultural system, cattle feedlots are a point in cattle production where animals are fed high-energy diets (called “finishing”) to grow out to an appropriate size and conditioning for slaughter processing (sometimes called “harvesting”), with an average time on feedlot of 6 months (2). There are several types of feedlots, our focus will be on those finishing feedlots that grow cattle from 700 pounds up to 1,300 pounds for the high quality, fresh/frozen beef market. Other types of feedlots may focus on finishing culled cows for ground beef or cooked beef product, feedlots that custom feed replacement females before they move back into breeding operations, or those backgrounding lighter weight calves that would then move to other feedlots for finishing. Knight (3) reports that, “feedlot operations with <1,000 head of cattle remain the majority, while feedlots in excess of 1,000 head encompass <5%.” Although larger feedlots make up a smaller proportion of all feedlots, they “market 80–85% of fed cattle, with feedlots of >32,000 head marketing 40% of fed cattle” (3). If 80–85% of fed cattle come from large feedlots, mass culling of these sites could rapidly devastate the beef cattle industry, which would reverberate along the entire production chain (4). Investigating how alternative strategies could be used on these sites is important to limit the impact of an FMD response within the US animal agricultural system. Specifically, can strategic depopulation, vaccination, and movement restriction be used within a single feedlot or will the virus “burn-through” the population too quickly to effectively use these response options in combination? To answer these questions, it is important to define disease transmission pathways on a feedlot that impact disease spread and response, including (i) where direct contacts occur animal movements on site (pen-to-pen) and how animals are moved through the feedlot, and (ii) who, what, and where the primary indirect contacts occur, with indirect contacts represented by the movement of feed-trucks, buyers, veterinarians, or pen-riders (personnel that enter pens, generally on horseback, to check cattle health daily) feed-trucks. There exist multiple design layouts and management styles for US feedlots (5, 6), presenting a challenge when modeling disease transmission and response on an individual site.

However, as a proof-of-concept experiment to allow critical questions to be explored, we can make assumptions about movements and pen types. Feedlot-related movements include cattle, vehicle, and human movements based on required structural features including roads or alleys for cattle movement and feed delivery. Pen types present on a feedlot include areas designated for receiving new cattle, treatment areas for sick or injured cattle (hospital pens), staging areas for shipping cattle, and home pens where cattle reside most of the time. These assumptions can then be clarified through solicited guidance from feedlot managers and agricultural extension educators to develop a range of expected behaviors across feedlots. This guidance provides details relevant to both direct movement, such as how often cattle are moved from a home pen to a hospital pen and how long they remain there, and indirect movement, such as how often veterinarians, feed-trucks, or pen-riders move around pens on a feedlot. These daily operational aspects of the feedlot influence the speed and extent of foot-and-mouth disease virus (FMDV) transmission across the site.

Consideration of the speed and extent of FMDV transmission subsequently influences the feasibility of the various response strategies available during an outbreak, dependent on the objective. Currently, the US has several response activities to consider during an outbreak, including mass depopulation (stamping-out), vaccination, movement restrictions, surveillance, and tracking (1). Stamping-out has multiple concerns ranging from the health of personnel, humane treatment of cattle, environmental protection associated with disposal, and the overall logistics of completing a large-scale depopulation. McReynolds et al. (7) conducted a Delphi survey of experts related to cattle production, health, pharmacology, toxicology, and cattle management to try to delineate the best method for depopulation on large cattle feedlots. Through this deliberation, the consensus was that there is not an optimal method to feasibly account for all concerns associated with a mass depopulation (8).

These expert discussions revealed significant challenges surrounding depopulation methods, ranging from environmental contamination to animal welfare. This led the panel to conclude that a large scale depopulation of a feedlot would have serious logistical issues for a rapid response and completion, while maintaining humane treatment of animals and safeguarding the environment after disposal (8). However, further concerns arise from the occurrence and management of asymptomatic carriers of FMDV that would endure in scenarios that do not include stamping out (9). Therefore, would it be logical to ask the questions, “How do we apply national level response strategies at the individual feedlot level?” and “Would this make biological sense for a response to a highly infectious pathogen?” When we consider depopulation of all susceptible animals on an infected premise, the depopulated animals can amount to thousands of feedlot cattle resulting in challenges that make it difficult to achieve response objectives. As such, alternative ways to apply response strategies on a large feedlot and the potential use of FMD vaccine should be evaluated. Disease modeling provides a tool to investigate alternative response strategies. This unique configuration of the national FMD InterSpread Plus (ISP) model provides an opportunity to refine a tool and assess the best methods for optimizing resources by understanding how the response activities on the feedlot influence loss of cattle and revenue.

Furthermore, several parameters in this tool can be adjusted through stakeholder input regarding the time required to vaccinate, depopulate, or move animals to slaughter. These parameters allow us to use the tool with specifications that fit different feedlot expectations and resource levels. Advantages could be realized such as, reducing disposal activities or the human resource and time commitment required by depopulation methods. Additionally, having access to a modeling tool specifically designed for large feedlots allows us to develop new response strategies and test their potential, prior to simulating strategies at the national level. ISP is a spatially explicit, stochastic state transition model, that provides a method to evaluate a distribution of outcomes within the US agricultural livestock system. This model framework has been used to configure disease spread simulations that guide outbreak policy and management (10–13). Subsequently, a novel reconfiguration of the current national FMD ISP model, using the parameterized infection dynamics and subject matter expert input, was a logical choice to test the potential of a new modeling tool.

To investigate the usefulness of this modeling tool and evaluate response strategies for a large feedlot our study focused on:

- Determining the disease dynamics of FMDV under “burn-through” conditions on the feedlot, in which the model will run with minimal response activities beyond biosecurity and movement restrictions.
- Evaluating how different response activities influence the epidemic curve of an FMD outbreak on a large feedlot.
- Performing a cost minimization analysis of strategies used across scenarios.

## 2. Methods

Many aspects of a feedlot must be considered when building a modeling tool that is focused on a single site versus a system of sites. One area is the spatial configuration, which is dependent on the space and purpose of the design. Feedlots often have animals housed in pens, lined up in rows, which can number in the thousands across a relatively small spatial extent, as in our sample feedlot site, which includes 54,790 head of cattle on a 1.64 km^2^ area. To accommodate these variations our configuration in ISP needed to account for the spatial arrangement of a large cattle population and movements within the feedlot site.

### 2.1. Model description

#### 2.1.1. Background

**Figure 1:**
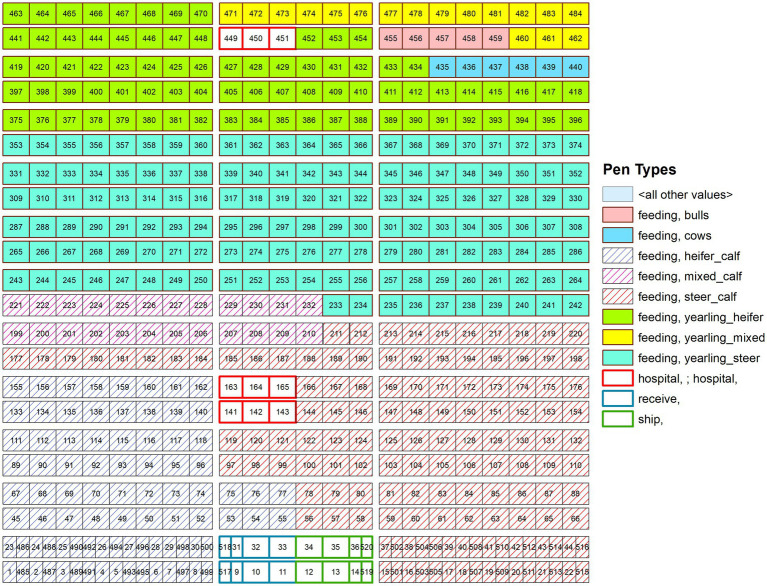
The layout of the feedlot representation in the InterSpread Plus model with a simplified design for the purpose of testing a reconfiguration of the ISP framework.

In the ISP framework the unit of operation is generally set to a whole farm as a single site production facility type such as a slaughter plant, a livestock market, cow-calf operation, dairy, or beef, etc. with movement occurring between these sites. However, in our configuration, we simulated a set of pens and therefore set each pen as a single unit within the ISP framework and developed movement patterns based on this conceptualization. For this simulation study we designed a feedlot loosely based on geolocated data from an existing site but reconfigured this to simplify the layout for our initial investigation (Figure 1). This provides necessary information to account for the distance between pens in the disease transmission simulation. The feedlot is a 54,790 head site with 520 pens divided into four categories: 495 feeding (home) pens, 8 receiving pens, 8 shipping pens, and 9 treatment (hospital) pens. Size and number of animals per pen varies by pen type with smaller animal per pen numbers in treatment areas and smaller sized pens for receiving and shipping areas. The majority of pens (61.6%) are designated as calf and yearling steers, while calf and yearling heifers take up the next highest proportion of pens (36.4%), and cows and bulls fill the remaining pens (2%) (Table 1). In the ISP framework we are not simulating individual animal movements and are therefore not modeling spread of disease based on one animal moving across a feedlot; as a stochastic state transition model transmission is based on the infection status of the units, in our case the pen. The simulation is run for 200 iterations for 365 days with a 60–365-day cutoff; dependent on the time required for response activities to be completed.

**Table 1:** Descriptive characteristics of the simulated feedlot.

Feedlot characteristics		Number of pens	Percentage of total pens	Number of animals
Total herd size	50,000	520	–	100 per pen^*^
Cattle types				
Steers < 700 lbs		154	0.302	15,100
Steers > 700 lbs		132	0.314	15,700
Heifers < 700 lbs		92	0.19	9,500
Heifers > 700 lbs		106	0.174	8,700
Cows		9	0.011	550
Bulls		2	0.009	450
Total feeding pens		495		
Specialty Pens^*^		25		

^*^Some variation due to specialty pens (shipping, medical treatment, and receiving).

Convergence was measured by calculating the cumulative median and percentage difference in the median from iteration to iteration and plotting these measures. We used the epidemic length because other outcomes such as Infected Premises were expected to always result in all pens being infected in the scenarios burn-through and harvest-NV. The epidemic length, on the other hand, did not have this same scenario-based effect and is therefore a better choice to test the convergence. Once the percent difference was below 5% the model was considered stable. Additionally, the median and the 90th percentile of iterations 50–150 and 50–200 were calculated and using a cutoff of 5% we calculated the percent difference between the two iterations sets for both the median and 90th percentile values, as done in Smith and Sanderson (14).

#### 2.1.2. Infectivity and transmission parameters

Parameters for transmission and pathogenesis are based on the FMDV serotype O due to the availability of data and global prevalence of this serotype. The risk for incursion into the United States remains high for this serotype, however, studies to develop parameters based on serotype A would be beneficial in the future. Initiation of the simulation begins with infection of a receiving pen on day 1 and detection occurring on day 7. The phases of infection were applied for a large cattle feedlot using values developed through experimental studies (15, 16). For instance, time to clinical signs was set to 5 days with a proportion of the specific animal type (calf-heifer, calf-steer, yearling-heifer, etc.) developing clinical signs each day until the 5th day is reached and clinical signs are exhibited for infected individuals. The maximum time of infectiousness was set to a Beta Pert distribution of 30, 34, 42 days. The pen-to-pen transmission parameters were developed using within-farm contact rates based on feedlot industry data and applied across all movements between pens. To simulate local area spread that is associated with distance between infected and susceptible pens via mechanisms that are difficult to trace, such as wildlife and aerosols, local spread parameters associated with a distance band of <1,000 m were incorporated (17) and were applied at a distance of 500 m out from an infected pen (Table 2). The four mechanisms of local area spread are defined as:

**Table 2:** The parameter values used in the feedlot model for the probability of transmission.

Model feature	Time (days)	Distance (m)	Probability of transmission	Notes
Pen-to-pen	1–23	–	0.511, 0.6168, 0.8189, 1.0, 1.0, 1.0 1.0, 1.0, 1.0, 1.0, 1.0, 1.0, 1.0, 1.0, 1.0, 1.0, 1.0, 0.9993, 0.9512, 0.511, 0.21, 0.21, 0.0	These values were set for each day to cattle movements between pens, such as movement to home, hospital, or shipping pens*
Off-site (processing)	1–23	–	0.0166, 0.0208, 0.3289, 0.9957, 1.0, 1.0, 1.0, 1.0, 1.0, 1.0, 1.0, 1.0, 1.0, 1.0, 1.0, 1.0, 1.0, 1.0, 1.0, 1.0, 0.3646, 0.0084, 0.0	These values were used for probability of transmission to the slaughter plant*
Pen-rider/Veterinarians	0, 0, 6, 11, 16, 21, 26, 31	–	0, 0.1, 0.2, 0.4, 0.5, 0.4, 0.2, 0.1	Taken from national model to represent personnel and veterinarians (indirect medium risk of transmission)
Feed-trucks	0, 0, 6, 11, 16, 21, 26, 31	–	0, 0.02, 0.04, 0.08, 0.1, 0.08, 0.04, 0.02	Taken from national FMD model to represent truck movement (indirect low risk of transmission)
Local spread 1 (pre-detection)	4	500	0, 0.007, 0.012, 0.012	The maximum distance from an infected pen and the probabilities that a neighboring farm will be infected. A probability value is used for each day from the time clinical signs appear, up to 4 days (17)
Local spread 2 (detected, pre-depopulation)	4	500	0, 0.00175, 0.003, 0.003	
Local spread 3 (depopulated, pre-disposal activities)	4	500	0, 0.000875, 0.0015, 0.0015	
Local spread 4 (detected, disposal not complete)	4	500	0, 0.000875, 0.0015, 0.0015	

*Values developed from data from the Beef Cattle Institute.

i. Local Area Spread 1: Detection on the feedlot has not occurred yet. (LS1).

ii. Local Area Spread 2: Detection has occurred, but depopulation has not begun (LS2).

iii. Local Area Spread 3: Depopulation has occurred, but the disposal activities are not complete (3-day window of C&D) (LS3).

iv. Local Area Spread 4: Detection has occurred, but disposal activities are not complete (LS4).

To calculate pathogen escape associated with local area spread (spread mechanisms that are difficult to trace) from the infected feedlot to off-site locations, there was an additional site located within the 500 m distance.

#### 2.1.3. Feedlot movement parameters

Simulating the movement of cattle, personnel, and equipment on a single feedlot is fraught with challenges, primarily related to the wide variety of circumstances, such as market volatility, seasonal variation in cattle volume, and disease incidence, which impact volume and frequency of cattle movements through a feedlot operation. However, management of cattle relies on animal welfare and biosecurity measures (5, 6, 18) that extend across both high and low volume periods. This understanding allows us to develop a model of disease transmission while incorporating movement of cattle, personnel, and equipment based on a general expectation of movement patterns.

To acquire a broader sense of these patterns and clarify the simplifying assumptions, we initiated a small focus group of researchers and extension specialists and asked targeted questions about the overall movement patterns on a feedlot. This allowed us to outline appropriate assumptions (Table 3) and define expected movement patterns (Table 4); with the understanding that the frequency of movements is heavily influenced by age class and season. For example, it would be expected that calf movements from a home pen to a treatment/hospital pen will be dramatically higher from August to November, but these same movements would be generally static for older yearling cattle throughout the year. Additionally, we decided on the assumption that all feeding pens would remain full throughout the simulation. This is based on the overarching goal of production to move animals on and off the feedlot in a synchronous manner. While this optimal situation is not always achieved, it is an appropriate simplifying assumption for our model simulations.

**Table 3:** Assumptions in the model by section type.

*Section*	Assumption	
Database *indirect vet movement*	Based on expert opinion veterinarians move primarily to a designated treatment location approximately one time per month. Due to biosecurity concerns and prevention measures, veterinarians generally do not physically enter other pens across the feedlot. In the treatment pens they train personnel and develop treatment protocols. Using a database eliminated over- and underestimation of veterinary visits in the ISP framework while representing our current understanding. Future work to better parameterize these movements could be informed by quantifying veterinary activities across a broad selection of feedlots.	
Database *Indirect feed truck*	Animals do not typically receive feed in shipping or receiving pens. Therefore, we assume that feeding pens and hospital pens are the primary pens for indirect feed truck contact. Feed-trucks traverse the feedlot 2 times per day, which is set up through a movement database.	
Database *Indirect pen-riders*	Shipping pens again are a short-term holding area and therefore not a site pen-riders would typically scan. Therefore, it is assumed that pen-rider activity will focus on feeding, treatment, and receiving pens for indirect contact. Treatment pens are included to account for pen-rider help in transferring animals between pens. Movements are simulated through a movement database with riders traversing the feedlot one time per day.	
Movement to hospital pens	Movements into the hospital pen come from feeding pens, there is a low probability of movement from a receiving pen due to injury in shipment. However, because this occurrence is infrequent the assumption is made that primary movements to a hospital pen will come from feeding pens. If infection exists in the receiving pen, without CS, upon arrival, this will be spread through the feedlot in a natural pattern of animal movement behavior. As mentioned previously receiving and shipping (staging area) pens are transitory, and animals are moved through in a manner to reduce stress. The goal of receiving pens is to conduct inbound activities (vaccinations, etc.) and move the animals to a home pen as quickly as possible. Shipping pens are a staging area to move animals off the feedlot.	
Surveillance	Passive surveillance on a feedlot is set to a constant 1, while visit delay is set to a constant 0, and delay to detection is set to BetaPert 0 0 1, which allows for some variability within detection. These parameters are used to reflect the expectation of personnel behavior and presence on the feedlot. Direct and Indirect surveillance are left in the model and reflect FAD PReP planning. These surveillance measures can be altered dependent on strategy implemented in the model.	

These assumptions are based on discussion with two distinct feedlots with differing management strategies. After discussion it is apparent that there is variability in management across feedlots but that there does exist some expected behavior regarding pen types. The assumptions below take these expectations into consideration.

**Table 4:** Parameter values used to simulate the movement patterns of cattle, personnel, and equipment on a single 50,000 head feedlot.

Movement	Number/Time Period	Description	Data source and parameter development
From cow-calf to receiving (primary movement)	Constant 1	Cattle are received daily onto the feedlot	Feedlot managers and extension specialists (Expert opinion)
Receiving pen to calf heifer pens	Poisson 0.171	A proportion of cattle movement expected to move calf heifers to calf heifer pens.	NAHMS 2011 (19)
Receiving pen to calf steer pens	Poisson 0.272	A proportion of cattle movement expected to move calf steers to calf steer pens.	NAHMS 2011 (19)
Receiving pen to calf mixed pens	Poisson 0.049	A proportion of cattle movement expected to move to a mixed pen of calf- heifers and-steers.	NAHMS 2011 (19)
Receiving to yearling heifer	Poisson 0.156	A proportion of cattle movement expected to move yearling heifers to yearling heifer pens.	NAHMS 2011 (19)
Receiving to yearling steer	Poisson 0.286	A proportion of cattle movement expected to move yearling steers to yearling steer pens.	NAHMS 2011 (19)
Receiving to yearling mixed pens	Poisson 0.048	A proportion of cattle movement expected to move to a mixed pen of yearling-heifers and-steers.	NAHMS 2011 (19)
Receiving to cow pens	Poisson 0.011	A proportion of cattle movement expected to move cows to cow pens	NAHMS 2011 (19)
Receiving to bull pens	Poisson 0.009	A proportion of cattle movement expected to move bulls to bull pens	NAHMS 2011 (19)
From feeding pens to treatment areas (primary movement)	Bulls/cows: Poisson 0.11 Calf pens: Poisson 2.44 Yearling pens: Poisson 1.55	Expected proportion of movements from each pen type to a treatment area	NAHMS 2011; derivation using weight class prevalence of Bovine Respiratory Disease (BRD) (19)
Movement	Number/Time Period	Description	Data source and parameter development
Treatment to calf-heifer, − steer, −mixed pens	Poisson 2.44	Movement into the treatment pens are matched with the outgoing movement	NAHMS 2011; derivation using weight class prevalence of Bovine Respiratory Disease (BRD) (19)
Treatment to yearling -heifer, −steer, −mixed pens	Poisson 1.55	Movement into the treatment pens are matched with the outgoing movement	NAHMS 2011; derivation using weight class prevalence of Bovine Respiratory Disease (BRD) (19)
treatment to cow or bull pens	Poisson 0.11	Movement into the treatment pens are matched with the outgoing movement	NAHMS 2011; derivation using weight class prevalence of Bovine Respiratory Disease (BRD) (19)
Consulting veterinarian to feedlot	Poisson 0.00365 Database	Veterinarians are expected to visit a feedlot ~1 time/month and enter a designated treatment area without entering other pens on the feedlot.	Expert opinion.
To shipping area from feeding pens (primary movement)	Cow/bull: Poisson 0.0106 Calf pens: Poisson 0.2615 Yearling pens: Poisson 0.2593	Proportion of movements out of feeding pens expected for each age group on the feedlot	Beef Cattle Institute (BCI) data for movement off a feedlot per week
From shipping area to slaughter plant	Poisson 0.857	Expected movement to a slaughter plant	Expert opinion explaining shipments off a feedlot occur 6 days a week.
Indirect feed truck	Poisson 940 Database	Expected that the feed truck visits each pen 2 times per day. This is set as a database source for movement.	Expert opinion
Indirect pen-riders	Poisson 460 Database	Expected to visit each pen once per day, certain pens are not visited by the pen-riders (see assumptions Table 2)	Expert opinion

Movements in our simulation focused on feed-trucks, pen-riders, veterinarians, and cattle movement that included, movement from receiving pens to home pens, home pens to treatment (hospital) areas, home pens to shipping, and shipping pens to an off-site location. We excluded any movement that was related to pen repair and maintenance due to lack of information and focused on daily activities related to health, welfare, and transition of cattle onto, within, and off the feedlot. Through our discussion it became clear that, on average, consulting veterinarians would not be expected to physically enter multiple pens across a feedlot, but instead would enter a treatment location where personnel are trained, and protocols are developed for animal treatment plans. This type of general knowledge allowed us to build our model with a primary expectation of where movements would occur, dependent on cattle age/type, personnel assignment, and equipment.

On the feedlot, roads provide access between the rows of pens for movement of cattle, personnel, equipment, and visitors such as buyers and veterinarians who are driven through for visual inspections of cattle (personal communication with feedlot managers). Movement to, within, and from the feedlot is influenced by the spatial configuration, which subsequently influences the spread of the pathogen. In our model we start by outlining what movements are important to the specific modeling objectives for a single feedlot. As such, our focus is on movement that occurs within the feedlot.

However, off-site movement, as a single movement type, is included as a geolocated site to calculate pathogen escape, which is the occurrence of a successful infectious movement of FMDV infected animals off the feedlot to another location. We made the assumption that this movement sent cattle to a single slaughter plant location because our simulation focuses on within feedlot transmission and response and because 98% of off-site movement from a feedlot ends at a slaughter plant (2). In addition, we included a single movement type onto the feedlot to evaluate disease spread after receiving infected animals and time to detection. For movements within the feedlot, we incorporated movement of feed-trucks, pen-riders, veterinarians, and cattle. These movements were subject to movement restrictions after FMD is detected dependent on their function. We recognized that certain movements such as feed-trucks and pen-riders would not be stopped during an outbreak because of the necessity for animal care and welfare. Parameters for indirect movements of pen-riders, veterinarians, and feed-trucks were based on those from the national level FMD model (15). Medium-level parameters were applied to pen-riders and veterinarians due to their direct movement into each pen or treatment pen, respectively, as opposed to feed-trucks that do not enter pens directly and therefore are defined as a low level transmission factor (Table 2). This allows us to explicitly simulate disease spread around each pen that becomes infected, with continued spread to pens across the feedlot based on direct and indirect movements that represent how the feedlot operates.

The frequency of each movement type was based on either data from the NAHMS 2011 report or expert opinion concerning the operational movements within a feedlot. The initial movement onto a feedlot occurs daily with multiple movements from receiving areas out to feeding pens per day. The proportion of movement out of receiving to feeding pens is based on the proportion of each cattle type (i.e., calf-, or yearling- steers and heifers, or cows and bulls) on our sample feedlot. Movement from these feeding pens to a treatment or hospital pen is based on the prevalence of Bovine Respiratory Disease (BRD), commonly referred to as shipping fever, and turnover rates for each cattle group. Prevalence was taken from the NAHMS 2011 report, grouped by weight class, those <700 lbs. (calf- steers and heifers) and those cattle >700 lbs. (yearling- steers and heifers, cows and bulls) (2).

Turnover rates were calculated from feedlot industry data. Using these data, we were able to estimate how many movements would occur to or from all treatment pens (9 total) for each cattle pen type (see Supplementary Tables S1–S3). Equipment and personnel movement was based on expert opinion from discussions with extension specialists and researchers. From this information we assumed that feed truck movement would occur 2 times per day, with the exclusion of receiving and shipping pens, while pen-riders are expected to enter each pen once per day with the exclusion of shipping pens. Veterinary movements are assumed to occur once per month with movement only occurring into treatment pens and off-site movements occur 6 days per week (parameters are described in Table 4).

Incorporation of these various movements allowed us to analyze each movements’ influence on the transmission patterns of FMDV within the feedlot. Studies have shown that indirect transmission can account for >40% of transmission (7, 20), requiring us to be able to delineate direct and indirect movements within the feedlot. Multiple pathways for indirect transmission exist on a feedlot from the various movements of personnel, equipment, and local spread. Local spread parameters are incorporated to simulate disease spread across pens and during cleaning and disinfection of depopulated areas. Direct transmission results from animal movements between receiving to home, home to hospital, and home to shipping pens.

#### 2.1.4. Model scenarios

Table 5 outlines the individual components of the scenarios investigated. Briefly, we simulated 6 strategies: (i) burn-through—managed “burn-through” which allowed the outbreak to spread through the feedlot, (ii) depopulation—a strict stamping-out scenario that implemented depopulation of the entire feedlot upon detection of FMDV, (iii) harvest—pens have vaccination implemented and then are allowed to recover from infection before being selected for harvest, (iv) harvest-NV - animals in the pens do not receive vaccination and are allowed to recover from infection before being selected for harvest, (v) firebreak—targeted depopulation of infected pens and adjacent pens with vaccination used to create firebreaks around infected pens, and (vi) firebreak-NV—a firebreak scenario that used targeted depopulation without vaccination. These scenarios were designed to evaluate how ISP can be configured to assess response strategies such as whether depopulation can be avoided or minimized on large cattle feedlots while achieving reduced outbreak duration and intensity.

**Table 5:** The single feedlot model scenario control measures and descriptions.

2020–2021 alternative strategies to stamping out on a single feedlot: scenario matrix						
Scenario	Stamping out	Firebreak		Selected harvest		Burn-through
Description	Depopulation of the feedlot, as a comparison model	Depopulation of infected pens and adjacent pens (either 1 or 2 rows) initial runs suggest longer outbreak with more depopulation		Apply a 100% movement restriction for 28–30 days, then use select-for-harvest set state to send pens to slaughter.		Managed outbreak with movement restrictions
Scenario tag	Depopulation	fireNV	Fire	Harvest-NV	Harvest	Burn-through
Stamping out	X					
Depopulation		X	X			
Send-to-slaughter				X	X	
Vaccinate			X		X	
Burn-through				X		X
Total model scenarios						6

#### 2.1.5. Control strategies

This model is based on the elements found in the national ISP FMD model (21). After the initial detection, a movement standstill was implemented to restrict any movements on and off the feedlot site, as well as movement restrictions for within site movement. Furthermore, after the initial detection, feedlot surveillance was initiated, with the expectation that surveillance relies heavily on passive surveillance, which is the observation of clinical signs by feedlot personnel. Additionally, cleaning/disinfection and disposal are initiated after depopulation and completed in 3 days. The use of control strategies, from the national model, were modified to fit a simulation for a single feedlot. For instance, movement restrictions were designed according to how cattle, personnel, and equipment move between pens on a feedlot in relation to when the first detection occurs on the feedlot (see Supplementary Table S4). Implementation of strategies such as depopulation and vaccination were adjusted to fit expectations for the number of pens that can be processed in 1 day. For instance, we assumed 500 animals can be vaccinated per day on a feedlot, which equates to ~5 pens dependent on pen type. As the outbreak continues vaccination increases to 8 pens/day. The speed in which feedlot management and outbreak response teams can vaccinate or depopulate may be an important area to explore using this tool. We acknowledge that mortality beyond depopulation activities will also occur during a real event, but do not explicitly model euthanasia due to humane reasons within this simulation. Any transmission of FMDV from these activities is accounted for in our indirect and local spread parameters.

Analysis of model output was completed in RStudio (22) using ggplot2 for visualization, and tidyverse, dplyr, and purr for analysis (23–25). Descriptive statistics were used to assess differences between control strategy activities (“burn-through,” depopulation, vaccination, and harvesting post outbreak) by evaluating the duration of outbreak, epidemic curve, movements associated with infections, and pathogen escape from the feedlot resulting from cattle shipped off site. During the analysis we found that the movements associated with the pen-rider and feed-trucks caused some minimal infections in pens that had been depopulated. The ISP framework does not allow us to restrict the database by specific pen characteristics, such as depopulated, which resulted in the need to correct the issue by removing the excess datapoints. This is appropriate because these movements to depopulated pens and subsequent infections would not occur on the feedlot due to the pens being empty; if left these would inflate our infection numbers.

For this initial investigation we have calculated the median, 25th, and 75th percentiles for comparing scenario outcomes in order to assess the concept of reconfiguration of the US national FMD ISP model to a single site. Further data was tabulated to assess the proportion of iterations resulting in the escape of FMDV off the feedlot (pathogen escape), the proportion of infections associated with specific spread mechanisms, proportion of detections by each surveillance mechanism, the median values for pens both vaccinated and depopulated, and the total number of pens vaccinated. These tests and summaries provide details about how the model represents FMDV transmission within a feedlot and how the different response strategies influenced the outbreak duration and magnitude.

To assess the feedlot level (on-farm) cost of the various strategies used in these scenarios, we used a cattle response budget calculator (see Supplementary Appendix 1) designed to calculate the costs associated with depopulation, disposal, cleaning and disinfection, and vaccination based on the time requirement per head of cattle for each activity category for cattle operations of varying types and sizes. Cost per item value was based on market values for labor, supply, and materials as determined through cost estimation methods similar to those used in the literature. Final cost estimates were compared to the literature (26–28) for consistency. In the calculator we selected a low-cost budget scenario that used captive bolt with euthanasia solution for depopulation and burial on-site for disposal. The cost estimate includes the labor, supplies and equipment needed to apply these controls in a timely manner, in compliance with state regulations. In comparison, we selected a high-cost budget scenario using dart gun tranquilizer with euthanasia solution for depopulation and landfill disposal. These two cost scenarios provide the extreme ends of the cost distribution to provide information about the cost associated with the different response activity combinations used in our simulation study, which cannot be generalized beyond this example. In scenarios using depopulation an indemnity value of $1355.00/hd USD (29) was used to calculate the total indemnity paid for the number of cattle depopulated. For scenarios that hold cattle for harvesting we used the value representing the lowest week market value from 2020 ($1178.87/hd) (30) (see Supplementary Tables S5, S6). Multiplying our epidemiological outputs (the number of cattle depopulated, held, and vaccinated) by the per head activity costs from the calculator, we calculated the feedlot response costs for each scenario.

#### 2.1.6. Model verification

The ISP model is a fully validated modeling framework (13, 31) that we have reconfigured through parameterization to a single site. Manipulating ISP to represent each pen within a single site rather than using individual farms across a landscape was the primary are of concern. To validate this conceptual aspect of the model movement across the site was evaluated and aligned with the expected movement patterns provided through subject matter expert solicitation, referred to as face validation (32) and parameter values are described in the Model Description under Feedlot Movement Parameters, The movement patterns were visualized through graphing the proportion of movement by type and using ArcMap to identify problems with the pattern across the site. These analysis tools allowed for adjustments to the model parameters to achieve the desired pattern. Verification of the underlying processes within the model were evaluated by assessing output and identifying model functionality (32). One area of concern was a minimal increase in infections from movements associated with pen-riders and feed-trucks to pens that had already been depopulated. ISP provides two options to represent movement between pens, by use of a movement database where each movement is known and pre-determined, or by simulating movements from probability distributions. We found that using a database for these movements resulted in the inability to stop movements once a pen was depopulated, which resulted in infectious spread to empty pens. By evaluating these infections, we were able to determine when this was occurring and remove the extra data points from the results that were used in analysis. The database method was still preferred because it allowed us to define the movement to each pen, compared to random movements generated from probability distributions that resulted in repeated movement to some pens and no movement to other pens, which is not an accurate representation of this movement. In addition to movement patterns, local spread within a feedlot was adjusted in a way to reflect fence line transmission. To accomplish this the radius was tested at various values (250, 500, 1,000 m) and transmission resulting from this spread mechanism was analyzed, which resulted in minimal differences at each distance. The distance of 500 m was chosen to represent the spread from pen to pen (Table 2).

The convergence criteria of <5% difference for the cumulative median of the epidemic length was achieved within 25 iterations in all scenarios (Supplementary Figures S1–S6). Furthermore, the comparison of the percent differences for the median and 90th percentile of iterations 50–150 and 50–200 was below 5% across all scenarios (Supplementary Table S7).

## 3. Results

After completing the verification process to ensure that the model was functioning, as expected, we ran multiple disease spread scenarios to assess transmission dynamics in this environment, which provides conceptual validity (33). We discuss these disease measures below to highlight the output from this model and for discussion of future development.

### 3.1. Factors of the epidemic curve

The characteristics of the epidemic curve provide our starting point for comparison of our model scenario outcomes. The duration of the epidemic (Table 6) calculated from the initial infection to the last day of infection was longest in the firebreak scenario with a median (25th, 75th percentiles) duration of 47 (38, 55) days. This scenario utilized both targeted depopulation of infected pens and adjacent pens and vaccination that extended from the edge of the depopulation zone to the remaining pens on the feedlot. The shortest epidemic duration occurred in the “burn-through” scenario (burn-through) with a median (25th, 75th) duration of 31 (25, 33) days (Table 6).

**Table 6:** Summary output of the epidemic and response activities for each scenario of the ISP single feedlot model that had 54,790 head of cattle in 520 pens.

Epidemic summary					Response summary				
Scenario	Epidemic duration (days)	Total pens infected	Peak day	Peak count of infected pens	Pens depopulated or harvested	Pens vaccinated	Pens depopulated and not infected	Pens vaccinated and depopulated	Pens not depopulated or infected
Burn-through	31 (30, 33)	520 (na)	18 (17, 20)	56 (53, 59)	0	NA	NA	NA	0
Depopulation	33 (31, 35)	390 (371, 407)	20 (19, 22)	39 (36, 42)	520 (na)	NA	130 (112, 149)	NA	0
Firebreak	47 (38, 55)	410 (392, 421)	20 (18, 22)	39 (36, 41)	453 (442, 461)	102 (95, 111)	106 (96, 123)	36 (28, 45)	67 (59, 78)
Firebreak-NV	34 (31, 37)	400 (385, 412)	20 (18, 21)	38 (36, 42)	520 (na)	NA	118 (105, 133)	NA	0
Harvest	35 (32, 39)	520 (na)	19 (17, 20)	54 (52, 58)	520 (na)	520 (na)	0 (max 1)^*^	520 (na)	0
Harvest-NV	32 (30, 34)	520 (na)	19 (17, 20)	56 (53, 59)	520 (na)	NA	0	NA	0

All values presented as median (25th, 75th). *In 9% of iterations one pen that was never infected was harvested; na—there was no variation across iterations partly due to scenario designs.

NA, not applicable for this scenario.

The magnitude of the epidemic is another measure we evaluated and is characterized by the peak day and count of infection and the total pens infected. Among our scenarios we found that those scenarios implementing depopulation (firebreak, firebreak-NV, depopulation) there was a delay in the peak day of infection compared to those not using depopulation (harvest, harvest-NV, and burn-through) with median (25th, 75th) ranges of 20 (18, 22) and 18–19 (17, 20), respectively (Table 6). Additionally, the peak count of infected pens (the highest number of infected pens on any given day) exhibited the same patterns between scenarios that depopulate versus scenarios that do not depopulate. In this instance, depopulation decreased the peak count of infected pens, while non-depopulation scenarios increase the peak count, with medians (25th, 75th) ranging from 38 to 39 (36, 42) and 54–56 (52, 59), respectively (Table 6).

The total/cumulative number of pens infected (Table 6) was reduced for the scenarios, firebreak-NV, firebreak, and depopulation, all of which implement depopulation efforts as part of the response strategy. The median number of pens infected was smallest for the depopulation scenario with a median (25th, 75th) of 390 (371, 407) infected pens. Whereas the burn-through and harvest-NV scenarios consistently had all pens (520) becoming infected, which was an expectation of the study design. The harvest scenario, which implements vaccination, had all pens become infected in 182 (91%) iterations, while 18 (9.0%) iterations resulted in all but one pen becoming infected.

### 3.2. Influence from response strategies

When we assessed the response strategies, we found that using a combination of depopulation and vaccination (firebreak) resulted in fewer depopulated pens with a median (25th, 75th) of 453 (442, 461) (Table 6). Additionally, we calculated the number of uninfected pens being depopulated and found that the highest number of depopulated uninfected pens occurred in the depopulation scenario with a median (25th, 75th) of 130 (112, 148) pens; while the fire scenario had the lowest number of depopulated uninfected pens with a median (25th, 75th) of 106 (96, 122). Interestingly, the firebreak scenario also had pens that were never infected or depopulated [67, (5, 78)] whereas all other scenarios resulted in all pens infected, depopulated, or both.

The number of pens vaccinated only applied to the firebreak and harvest scenarios. A comparison of the total pens vaccinated between these two is inconsequential because the harvest scenario is designed to vaccinate all pens, while the firebreak scenario vaccinates after targeted depopulation, reducing the number of total pens vaccinated in this scenario. However, we did evaluate the proportion of infections that occurred after vaccine deployment. In the firebreak scenario, the median (25th, 75th) proportion of pens infected post vaccine deployment was 24% (16.7, 31.3) of pens that were infected and vaccinated, while the remaining were infected prior to vaccination (Table 7). In the harvest scenario, the median proportion of pens infected post vaccination was 5% (4.0, 6.0) of the pens that were infected and vaccinated, with the remaining becoming infected prior to vaccine deployment (Table 7).

**Table 7:** The median counts and proportion of pens vaccinated and infected post vaccination.

Median values of pens vaccinated and infected			
Scenario	Total pens vaccinated and infected	Median number of pens infected post-vaccination	Median proportion of pens infected post-vaccination (%)
Firebreak	27 (21, 34)	6 (4, 9)	24 (16.7, 31.3)
Harvest	520 (NA)	27 (22, 31)	5.0 (4.0, 6.0)

### 3.3. FMDV transmission mechanisms within the feedlot

Three mechanisms that resulted in > 5% of infectious spread per mechanism consistently showed up across all scenarios and included, local area spread (1, 2, and 4), movement of pen-riders, and movement of feed-trucks. LS3 is not a factor in the harvest, harvest-NV, or burn-through scenarios because depopulation and disposal activities are not used in these designs, voiding LS3 as a transmission mechanism. LS1 accounts for most of the transmission across all scenarios with a median (25th, 75th) range of 35.7–38.2% (32.9, 40.7). The median range of LS2, 3, 4 are: (i) 29–32% (27, 33.8), (ii) 1.7–2.0% (1.0, 2.7), (iii) 15.5–16.3% (14.6, 17.8), respectively. The feed-truck and pen-rider had medians of 3.2–4.1% (2.6, 4.7) and 8.0–10.0% (7.3, 10.8), respectively (Supplementary Figures S7–S12).

### 3.4. Pathogen escape off the feedlot

Pathogen escape, occurrences of infectious spread from the feedlot to off-site locations, is an important aspect of the model output. We were able to capture the mechanism of dispersal and the overall proportion of iterations within each scenario that resulted in infectious movement off the feedlot. Overall, >92% of iterations in each scenario resulted in pathogen escape. Most of these, >41% in each scenario, were due to local spread that occurred after detection of FMDV but prior to any depopulation activities. In >20% of iterations pathogen escape resulted from local spread prior to detection of FMDV and another >20% was due to local spread that occurred after detection but prior to cleaning and disinfection activities. Pathogen escape, due to the movement of infected animals (i.e., an infected pen sent to slaughter) from within the feedlot to a slaughter plant, occurred in ≤1.5% in each scenario (Table 8), the highest proportion occurring in the firebreak scenario with 1.5% and the lowest proportion occurring in the depopulation and harvest-NV scenarios with both at 0.5%. There was no pathogen escape due to movement to slaughter for either the firebreak-NV or harvest scenarios.

**Table 8:** The proportion of iterations that resulted in an infectious movement off the feedlot by either local spread or movement of cattle to the slaughter plant.

Proportion of iterations with pathogen escape off the feedlot						
Scenario	Total iteration escape	Slaughter escape	LS1	LS2	LS3	LS4
Burn-through	0.995	0.01	0.265	0.495	NA	0.235
Depopulation	0.975	0.005	0.27	0.42	0.035	0.25
Firebreak	0.925	0.015	0.21	0.415	0.015	0.285
Firebreak-NV	0.95	NA	0.205	0.425	0.025	0.295
Harvest	0.98	NA	0.255	0.43	NA	0.295
Harvest-NV	0.995	0.005	0.295	0.5	NA	0.2

The movement or transmission mechanisms are Slaughter Escape: movement off the feedlot to a slaughter plant, this can occur prior to movement restrictions or during select for harvest activities. Local Spread 1: Detection on the feedlot has not occurred yet. (LS1); Local Spread 2: Detection has occurred, but depopulation has not begun (LS2); Local Spread 3: Depopulation has occurred, but the disposal activities are not complete (3-day window of C&D) (LS3); Local Spread 4: Detection has occurred disposal activities are not complete (LS4).

### 3.5. Feedlot response cost minimization analysis

Using the cattle response budget calculator allowed us to evaluate the cost associated with the number of cattle depopulated, vaccinated, or held for harvest in our simulation scenarios (Table 9). Through this analysis we found that in both the low- and high-cost budget scenarios (Supplementary Tables S5, S6), the simulations that held cattle for processing post-outbreak resulted in the lowest cost for feedlot response activities by > $12 million USD in the low-cost budget scenario and nearly $20 million in the high-cost budget scenario, when compared to the depopulation and firebreak-NV scenarios. In the comparison between the harvesting scenarios to the fire scenario, which used targeted depopulation with vaccination, we found a cost reduction of >$10 million for the low-cost budget and > $17 million for the high-cost budget. The firebreak simulation had the third lowest cost with a cost reduction of $1.5 million for the low-cost budget and $2.5 million for the high-cost budget scenarios compared to the depopulation and firebreak-NV scenarios (see Supplementary Table S8). The highest cost scenarios were the depopulation and firebreak-NV, which are essentially the same because the targeted depopulation without vaccination scenario results in a full depopulation of the feedlot site.

**Table 9:** The cost of each scenario under a low- and high-cost budget that shows the cost of response activities on a single feedlot with and without estimated indemnity ($1,355/hd) and harvest ($1178.87/hd) value.

Low-cost budget			High-cost budget		
	Cost w/o indemnity or market low	Total cost		Cost w/o indemnity or market low	Total cost
Harvest-NV	177,858.18	64,768,145.48	Harvest-NV	177,858.18	64,768,145.48
Harvest	840,370.02	65,430,657.32	Harvest	840,370.02	65,430,657.32
Firebreak	3,572,636.16	76,527,337.16	Firebreak	9,629,627.76	82,584,328.76
Firebreak-NV	3,808,733.43	78,049,183.43	Firebreak-NV	10,803,786.83	85,044,236.83
Depopulation	3,808,733.43	78,049,183.43	Depopulation	10,803,786.83	85,044,236.83

## 4. Discussion

The objective of this work was to evaluate whether the ISP framework could be used to develop a modeling tool to evaluate within-farm transmission and response strategies for an FMD outbreak on a single large feedlot in the US. This is the first step toward development of a single site ISP application that could provide increased utility and real-time flexibility with further testing and model parameterization. The stochastic and spatial features of the ISP framework offer an optimal modeling environment for investigating this complex issue. The outcomes are a distribution of possibilities that better express the inherent variability within biological systems (12).

Our model results (Table 6) suggest that the duration of FMDV transmission through the feedlot is the shortest in the “burn-through” scenario, while the combination of targeted depopulation and vaccination (firebreak) results in the longest epidemic duration. In Cabezas et al.’s simulation study with intervention scenarios that included, restricted movement between hospital pens and home pens, barrier depopulation, and targeted depopulation compared to a non-intervention scenario suggested a longer epidemic duration overall compared to the ISP simulations in this study (34). This difference could be a result of the intervention strategies modeled, feedlot size and structure, and model development. The simulations in ISP focused on multiple intervention strategies including a complete depopulation and vaccination on a feedlot with >50,000 head of cattle, using parameters for the rate of depopulation and vaccination that fluctuated with expected resource availability. Findings from this study suggests that limiting response activities increases the speed of transmission across the feedlot while implementation of various activities will slow transmission across the site and reduce the overall number of pens infected. This reduction in infected pens could influence the amount of virus present and reduce the potential for transmission on- and off-site.

In our simulation study we found varying levels of difference in the median epidemic length depending on the response strategies used, such as sending cattle to harvest and using vaccination or targeted depopulation without vaccination. This suggests that there will be critical nuances to consider dependent on the outbreak situation and desired outcomes that could be used without an expectation of altering the epidemic duration. Interestingly, the harvest scenario showed a reduction in the median epidemic duration of 12 days (Table 6) compared to the median epidemic duration of the firebreak scenario. Again, how to interpret the relevance of these results will depend on the response objectives and other factors, such as resource availability, limiting depopulation, and implementing slaughter processes to decrease animal loss.

The firebreak scenario, which uses both targeted depopulation and vaccination, created a situation where there was a slow burn of FMDV transmission through the feedlot compared to all other scenarios. If the objective is to increase the number of uninfected pens, then targeted depopulation in combination with vaccination (firebreak) showed potential, as the only scenario to leave pens uninfected and not depopulated with a median (25th, 75th) of 67 (59,78) pens. However, its success is dependent on vaccine availability and the speed at which cattle can be vaccinated. This is comparable to Cabezas et al.’s findings, which used barrier depopulation (like our targeted depopulation label), and was suggestive of an increasing proportion of simulations where transmission was interrupted as the rate of depopulation increased (34). Understanding the extent of vaccination use in each scenario provides context for evaluating the usefulness of each strategy individually and in combination with other response strategies, like depopulation. The use of vaccination prior to sending animals to slaughter in the harvest scenario resulted in one uninfected pen in 9% of iterations, suggesting that the. Speed of vaccination deployment and biosecurity measures should be further explored in the ISP configuration to determine if transmission could be reduced to a beneficial level.

The response strategies implemented are dependent on the outbreak situation, which is informed by epidemiological factors, public acceptance, industry concerns, and animal health and welfare (1, 35). If the objective is to reduce the susceptible population and the amount of virus, scenarios using depopulation and/or vaccination may be optimal. However, if the objective is to reduce the use of depopulation and increase transmission across the feedlot to achieve a quicker recovery state for the movement of animals to slaughter, scenarios that limit depopulation or vaccination could be more favorable.

One way to understand the differences and potential of each scenario design is to develop a ranking scheme. We did not develop a validated ranking scheme but to illustrate the concept in a purely hypothetical manner, we can think of each outcome as categories to be assigned a value and quantify the strategies used to help determine which strategy is best in a particular situation. For example, we may have a set of objectives that aim to reduce the duration, total pens infected, and total pens depopulated, while increasing pens not infected or depopulated (saved pens). Developing a ranking scheme for these outcomes would show which strategy falls closest to the objectives. This would be an additional tool for planners and responders to incorporate into their guidance resources.

Furthermore, these results lead to questions regarding the combined use of limited resources. For instance, the firebreak scenario resulted in a proportion of pens being saved, while extending the epidemic to our longest duration of 47 (38, 55) days. Does this translate to an economic benefit when both targeted depopulation and vaccination are used on a single feedlot? Feedlot production objectives include economic, health, nutrition, and animal welfare considerations, and added stress from illness increases the demand for labor and the cost of health management for the feedlot for non-FADs like bovine respiratory disease (36). For depopulation alone, there are multiple pharmacological agents available but concerns arise about how practical they are when considering the logistics of moving cattle from a chute after death to a disposal site (8). Further, McReynolds and Sanderson (8) study elicited veterinarians’ and feedlot managers’ to gain insight regarding concerns about animal welfare and public perception. They found that depopulation methods that do not include chutes or alleys (e.g., shooting cattle in lanes or pens) had higher risks of negative public perception or animal welfare challenges. In South Africa, an outbreak of FMD in a feedlot was addressed with a vaccination policy rather than stamping-out due to logistical concerns about depopulating and disposing of over 14,000 cattle and 2,000 sheep in a small area (37). This illustrates the critical risk factors associated with insufficient labor, equipment and supplies for timely carcass disposal and the need to minimize the risks of predation and further spread of disease by quickly removing and disposing of carcasses from infectious animals (8).

To answer questions about the economic aspects of the strategies in this study, we used a feedlot response cost minimization analysis, which showed that the lowest costs resulted from the harvest and harvest-NV scenarios, both of which hold cattle for post-outbreak processing. Evaluating both the epidemiology and the economic consequences the harvest and harvest-NV scenarios reduce the duration of the outbreak, use of depopulation, and the single-farm response costs associated compared to other scenarios that implemented targeted depopulation and vaccination. The harvest and harvest-NV scenario did differ from the scenarios implementing depopulation and/or vaccination regarding epidemic duration, peak day and count of infected pens, and the total number of pens infected. These harvest scenarios had shorter epidemic durations, earlier peak day of infection and higher numbers of infected pens both at the peak and cumulatively. This suggests that the FMDV spread through the feedlot quicker than when additional response activities were used, while allowing for the feedlot to avoid using mass depopulation. Couple that with a lower overall cost, these scenarios offer an alternative line of investigation for preparing for a potential incursion of FMD in the US agricultural livestock sector. This is one example of how the reconfiguration of ISP to a single feedlot site could help inform outbreak response planners and responders.

Overall, our epidemiological and feedlot response cost minimization analyses illustrates that there are potential alternatives to mass depopulation on large feedlots that require further investigation. The next step in this evaluation would be to test the scenarios on other feedlot structures and compare the model output and cost minimization analysis. The economic consequences could be expanded in future research by considering the national agricultural producer and consumer impacts under the best scenario from this analysis, particularly under varying levels of bilateral trade partner and domestic consumer response. The response by processors, retailers, and trade markets will be crucial to determining potential benefits from alternatives to depopulation.

Additional considerations that need to be addressed, include the feasibility of processors to take recovered and/or vaccinated cattle, WOAH mandatory waiting periods, the extent of trade embargoes by major markets for livestock and beef products, domestic consumer response, the impact of carrier cattle within the recovered population, the potential for pathogen escape during the hold time, and further evaluation of costs over the length of time the cattle are held on site. While most of these considerations are not within the scope of this study, we did find that pathogen escape to the processor was low with 0 and 0.5% of iterations resulting in pathogen escape for harvest and harvest-NV, respectively. Furthermore, across all scenarios, pathogen escape to a neighboring site occurred in >92% of iterations. This suggests a critical control point to consider when holding infected cattle on-site to reduce mass depopulation.

Model results indicated that the largest amount of pathogen escape resulted from transmission of FMDV off the feedlot through indirect routes related to local spread, suggesting that biosecurity is critical in any of the tested response strategies (Table 8). Our outcome suggests that biosecurity at the periphery of the farm will be a critical control point to limit the potential spread off -site. Considerations will also need to be given to potential contacts with feral swine or susceptible wildlife species (white-tailed deer) at the periphery of the farm site (38, 39). Within the farm site three mechanisms of spread consistently occurred across scenarios including, local spread (1, 2, and 4), feed-trucks, and pen-riders. Movements of animals between pens had limited impact on disease transmission but within our model we assume biosecurity measures and movement restrictions of animals are implemented on all pens.

However, due to the necessity to move personnel and equipment, for animal care and welfare, pen-riders and feed truck movements were not restricted. This could be an area that we focus biosecurity measures to potentially limit spread by implementing movement patterns and flows through an infected feedlot. Although, it is important to point out that the proportion of transmission resulting from personnel and equipment was ≤10 and 5% for pen-riders and feed-trucks, respectively. Local spread accounted for >80% of all within feedlot transmission in all scenarios, suggesting that a focus on cross pen transmission would be a good focus for control efforts, while limiting pen to pen contact would be critical to reduce transmission within the site.

On the other hand, the objective may be to increase the speed of virus transmission when managing a controlled outbreak that aims to either depopulate at a slower pace or harvest feedlot animals once they have recovered. In this type of instance, spread off the feedlot would remain the critical biosecurity control point while spread within the feedlot would be managed to minimize illness and discomfort and to support recovery of infected animals. It is important to acknowledge that measures that reduce the potential for the virus to spread to neighboring farm sites or processing plants would be critical in any situation, whether we are depopulating the site to reduce the infected, exposed, and susceptible population or aiming to reduce depopulation and economic impacts through a variety of response scenarios (40, 41).

Taken as a whole, the outcomes from this simulation study illustrated that the ISP framework can be configured to investigate within farm FMDV transmission and response strategies. This tool provides proof of concept that the ISP model can be reconfigured to explore response scenarios with specific goals in mind, to gain understanding about the possible outcomes from different emergency preparedness activities during an FMD outbreak. These scenarios cannot be generalized beyond the scope of this simulation study because they were designed to assess the potential to reconfigure ISP to a single site, not to answer specific questions based on response objectives. The feedlot configuration and movement patterns are specific to this simulation and therefore do not allow for generalization to other feedlot structures with different movement patterns. Scenarios with low, moderate, and high vaccination rates, vaccine availability and prioritization among different susceptible species and animal production types, or depopulation capabilities will help determine if these strategies are useful. Ultimately, helping to determine how vaccination is deployed or how depopulation is used. While the tool does provide flexibility in response design, it is important to understand the limitations.

Currently, the model is based on a single feedlot structure, but we know that feedlot designs vary tremendously. While we set our movement to expected patterns, these also vary across feedlots. However, by using a general layout and expected movements based on industry data on feedlot operations, endemic disease management, and animal care requirements, this initial design is useful to explore feedlot response strategies. Increasing our knowledge and data sources about feedlot structures and movements will improve our design and allow us to explore several feedlot layouts. Additionally, the parameter estimates are based on FMDV serotype O, which presents a high risk for incursion into the United States, however, studies to develop parameters based on serotype A would be beneficial in the future. Another area to maintain awareness of, is the presence of asymptomatically infected cattle including carriers and neoteric subclinical animals, especially when implementing scenarios that aim to keep cattle through their productive lives (42–44). The model does not explicitly include this population and planning decisions that use these outcomes would need to consider the consequences of carriers in a population of vaccinated or recovered cattle (9).

Additionally, we set our vaccination, depopulation, and movement off-site to a moderate or low level to gain general insight about the model function. These parameters are sections of the model that can be investigated through either internal or stakeholder motivated questions. In either circumstance, we can evaluate topics (and model input parameters) such as the time it takes to depopulate, vaccinate, or harvest and determine where the largest benefit is achieved and provide targeted output for response planning. Furthermore, we chose not to put limitations on the availability of the vaccine or resources to complete these activities beyond the time constraint. However, this tool expands our ability to continue to test, evaluate, and find optimal response options that reduce the impact of an FMD outbreak on the US agricultural livestock sector. A sensitivity analysis, to investigate disease transmission parameters on a single feedlot versus farm-to-farm spread in ISP, could provide further insight to the underlying variability of the disease process and should be evaluated as development of this model continues. Due to constraints at the time of publication this was not completed but will be important in future work.

## 5. Conclusion

Global movement of people, animals, and animal products requires that we continue to study ways to prevent, control, and eliminate foreign animal disease threats to protect the US livestock sector. Research and development of new tools and methods allow us to evaluate outbreak scenarios and response activities to increase our understanding of potential alternatives to current disease preparedness plans. This includes the ability to evaluate different response strategies for complex situations during an FMD outbreak, such as large cattle feedlots. The tool described herein allows us to both explore questions on this topic and consider these activities at the individual feedlot level from disease control and single feedlot response cost perspectives. This effort targeted livestock stakeholders, policy makers, and response planners to ask pertinent questions about the feasibility of various strategies that can be implemented on a large feedlot when stamping out is not a viable option. Economic analysis, specifically cost minimization, was used to determine best-case strategies from this individualized tool that can then be applied at the national level using the national FMD model. This economic analysis would be best complemented by a full market analysis, which would also account for the implications of vaccination and limited depopulation on potential trade bans by beef trading partners. Although, testing best-case strategies in the national model is beyond this work, these results can be expanded to determine if the best-case strategies hold at the national level. Evaluation of response strategies at individual premises and national levels will continue to advance our ability to prepare for, respond, and protect US agriculture in the event of an FMD outbreak.

## Data availability statement

The original contributions presented in the study are included in the article/Supplementary material, further inquiries can be directed to the corresponding author.

## Author contributions

SM contributed to conception and led development of the single feedlot model (sfm) design, completed model analysis, wrote, and edited the manuscript. LH contributed to the conception and development of the sfm design, reviewed the analysis, and edited the manuscript. CR contributed to the conception and development of the sfm design, reviewed the analysis, and edited the manuscript. AH contributed to the conception and development of the sfm design, provided cost minimization calculator, reviewed analysis, and contributed to writing and editing manuscript. AD contributed to the conception of the sfm design, reviewed the sfm design, and edited the manuscript. JA contributed to the conception of the sfm design, reviewed the sfm design, and edited the manuscript. TB contributed to the development, reviewed the sfm design, and edited the manuscript. All authors contributed to the article and approved the submitted version.

## Funding

This research was supported in part by an Interagency Agreement between CEAH/APHIS/USDA and FADRU/ARS/USDA. SM was a fellow within the Agricultural Research Service (ARS) Research Participation Program administered by the Oak Ridge Institute for Science and Education (ORISE) through an interagency agreement between the U.S. Department of Energy (DOE) and the U.S. Department of Agriculture (USDA). ORISE was managed by ORAU under DOE contract number DE-SC0014664. All opinions expressed in this manuscript are the author’s and do not necessarily reflect the policies and views of USDA, DOE, or ORAU/ORISE.

## Conflict of interest

The authors declare that the research was conducted in the absence of any commercial or financial relationships that could be construed as a potential conflict of interest.

## Publisher’s note

All claims expressed in this article are solely those of the authors and do not necessarily represent those of their affiliated organizations, or those of the publisher, the editors and the reviewers. Any product that may be evaluated in this article, or claim that may be made by its manufacturer, is not guaranteed or endorsed by the publisher.

## References

[1] USDA. Foot-and-mouth disease response plan. The Red Book National Preparedness and Incident Coordination Center (2020).

[2] USDA. Feedlot 2011 part I: management practices on U.S. feedlots with a capacity of 1,000 or more head. Fort Collins, CO (2011).

[3] Knight R. (2020). U.S. cattle production. Sector at a glance. Available at: https://www.ers.usda.gov/topics/animal-products/cattle-beef/sector-at-a-glance/

[4] Hardham JM Krug P Pacheco JM Thompson J Dominowski P Moulin V. Novel foot-and-mouth disease vaccine platform: formulations for safe and DIVA-compatible FMD vaccines with improved potency. Front Vet Sci. (2020) 7:554305. doi: 10.3389/fvets.2020.554305, PMID: 33088833 PMC7544895

[5] Euken R. Doran B. Clark C. Shouse S. Ellis S. Loy D. Schulz L. (2015). Beef feedlot systems manual. Iowa State University Extension and Outreach.

[6] USDA. (2011). FAD PreP beef feedlot industry manual. Produced by the Center for Food Security and Public Health, Iowa State University of Science and Technology, College of Veterinary Medicine with the U.S Department of Agriculture Animal and Plant Inspection Service.

[7] McReynolds SW Sanderson MW Reeves A Hill AE. Modeling the impact of vaccination control strategies on a foot and mouth disease outbreak in the Central United States. Prev Vet Med. (2014) 117:487–504. doi: 10.1016/j.prevetmed.2014.10.005, PMID: 25457133

[8] McReynolds SW Sanderson MW. Feasibility of depopulation of a large feedlot during a foot-and-mouth disease outbreak. JAVMA. (2014) 244:291–8. doi: 10.2460/javma.244.3.291, PMID: 24432961

[9] Yadav S Delgado AH Hagerman AD Bertram MR Moreno-Torres KI Stenfeldt C. Epidemiologic and economic considerations regarding persistently infected cattle during vaccinate-to-live strategies for control of foot-and-mouth disease in FMD-free regions. Front Vet Sci. (2022) 9:1026592. doi: 10.3389/fvets.2022.1026592, PMID: 36337179 PMC9632437

[10] Gibbens JC Sharpe CE Wilesmith JW Mansley LM Michalopoulou E Ryan JBM. Descriptive epidemiology of the 2001 foot-and-mouth disease epidemic in Great Britain: the first five months. Vet Rec. (2001) 149:729–43. doi: 10.1136/vr.149.24.729 11808655

[11] Hagerman AD Johnson KK Holmstrom LK Roigney C Boyer T Schoenbaum M. Saving our bacon without hamstringing the industry: sensitivity of economic losses to post-outbreak management of foot-and-mouth disease vaccinated animals in a simulated US outbreak In: Paper Presented at the Agricultural and Applied Economics Association Annual Meeting. Washington, DC (2018)

[12] Morris RS Wilesmith JW Stern MW Sanson RL Stevenson MA. Predictive spatial modelling of alternative control strategies for the foot-and-mouth disease epidemic in Great Britain, 2001. Vet Rec. (2001) 149:137–44. doi: 10.1136/vr.149.5.137, PMID: 11517981

[13] Stevenson MA Sanson RL Stern MW O’Leary BD Sujau M Moles-Benfell N. InterSpread Plus: a spatial and stochastic simulation model of disease in animal populations. Prev Vet Med. (2013) 109:10–24. doi: 10.1016/j.prevetmed.2012.08.015, PMID: 22995473

[14] Smith MR Sanderson MW. Modeled impacts of rapid and accurate cattle tracing in a Foot-and-Mouth Disease outbreak in the US. Prev Vet Med. (2023) 215:105911. doi: 10.1016/j.prevetmed.2023.10591 37084632

[15] USDA. (2013). Parameters used to simulate the spread of FMD in Texas using the north American animal disease spread model (NAADSM) for use in FMD response workforce requirement estimates. Available at: https://www.aphis.usda.gov/animal_health/nahms/modelling/downloads/NAADSM_TX.pdf

[16] Yadav S Stenfeldt C Branan MA Moreno-Torres KI Holmstrom LK Delgado AH. Parameterization of the durations of phases of foot-and-mouth disease in cattle. Front Vet Sci. (2019) 6:263. doi: 10.3389/fvets.2019.00263 31448297 PMC6696987

[17] Sanson RL Stevenson MA Moles-Benfell N. Quantifying local spread probabilities for foot-and-mouth disease In: Paper Presented at the 11th International Symposium on Veterinary Epidemiology and Economics. Cairns (2006)

[18] Harner JP III Murphy JP. Planning cattle feedlots. Kansas State University (1998).

[19] USDA. Feedlot 2011 part IV: health and health management on U.S. feedlots with a capacity of 1,000 or more head. Fort Collins, CO (2011).

[20] Bravo de Rueda C de Jong MC Eble PL Dekker A. Quantification of transmission of foot-and-mouth disease virus caused by an environment contaminated with secretions and excretions from infected calves. Vet Res. (2015) 46:43. doi: 10.1186/s13567-015-0156-5, PMID: 25928658 PMC4404111

[21] Boyer T Burdett C Delgado AH Garza S Hagerman AD Hillberg Seitzinger A. Development of a national model for foot-and-mouth disease in the United States In: Paper Presented at the Open Session of the Standing Techinical and Research Committees of the EuFMD. Cavtat (2014)

[22] RStudioTeam. RStudio: integrated development for R. Boston, MA (2020) Available at: http://www.rstudio.com/.

[23] Wickham H. ggplot2: elegant graphics for data analysis. New York, NY: Springer-Verlag (2016).

[24] Wickham H Averick M Bryan J Chang W McGowan LD Francois R. Welcome to the tidyverse. J. Open Sourc. Softw. (2019) 4:1686. doi: 10.21105/joss.01686

[25] Wickham H. Francois R. Henry L. Muller K. (2018). dplyr: a grammar of data manipulation. R Package version 0.7.6. Available at: https://dplyr.tidyverse.org, https://github.com/tidyverse/dplyr

[26] Carpenter TE O’Brien JM Hagerman AD McCarl BA. Epidemic and economic impacts of delayed detection of foot-and-mouth disease: a case study of a simulated outbreak in California. J Vet DIagn Invesi. (2011) 23:26–33. doi: 10.1177/104063871102300104, PMID: 21217024

[27] Deotte JRE Deotte IRE. Considerations for management of livestock during an infectious animal disease incident as an alternative to massive carcass disposal using foot-and-mouth disease in beef cattle feedlots as an example In: Paper Presented at the International Symposium on Air Quality and Manure Management for Agriculture. Dallas, TX (2010)

[28] Elbakidze L Highfield L Ward M McCarl BA Norby B. Economics analysis of mitigation strategies for FMD introduction in highly concentrated animal feeding regions. Rev Agric Econ. (2009) 31:931–50. doi: 10.1111/j.1467-9353.2009.01477.x

[29] USDA-FSA. (2021). Disaster assistance, livestock indemnity program. Available at: https://www.fsa.usda.gov/Assets/USDA-FSA-Public/usdafiles/FactSheets/livestock_indemnity_program_lip-fact_sheet.pdf

[30] LMIC. (2021). Livestock marketing information center. Available at: https://www.lmic.info/

[31] Sanson RL Harvey N Garner MG Stevenson MA Davies TM Hazelton ML. Foot and mouth disease model verification and ‘relative validation’ through a formal model comparison. Rev Sci Tech. (2011) 30:527–40. doi: 10.20506/rst.30.2.2051 21961223

[32] Garner MG Hamilton SA. Principles of epidemiological modelling. Rev Sci Tech. (2011) 30:407–16. doi: 10.20506/rst.30.2.2045 21961213

[33] Reeves A Salman MA Hill AE. Approaches for evaluating veterinary epidemiological models: verification, validation and limitations. Rev Sci Tech. (2011) 30:499–512. doi: 10.20506/rst.30.2.2053, PMID: 21961221

[34] Cabezas AH Sanderson MW Volkova VV. Modeling intervention scenarios during potential foot-and-mouth disease outbreaks within U.S. Beef Feedlots Front Vet Sci. (2021) 8:559785. doi: 10.3389/fvets.2021.559785, PMID: 33665214 PMC7921729

[35] Barnett PV Geale DW Clarke G Davis J Kasari TR. A review of OIE country status recovery using vaccinate-to-live versus vaccinate-to-die foot-and-mouth disease response policies I: benefits of higher potency vaccines and associated NSP DIVA test systems in post-outbreak surveillance. Transbound Emerg Dis. (2015) 62:367–87. doi: 10.1111/tbed.12166, PMID: 24112127

[36] Edwards TA. Control methods for bovine respiratory disease for feedlot cattle. Vet Clin North Am Food Anim Pract. (2010) 26:273–84. doi: 10.1016/j.cvfa.2010.03.005 20619184

[37] Bruckner GK Vosloo W Du Plessis BJA Kloeck PELG Connoway L Ekron MM. Foot and mouth disease: the experience of South Africa. Rev Sci Tech Off Int Epiz. (2002) 21:751–64. doi: 10.20506/rst.21.3.1368 12523712

[38] Brown VR Bevins SN. Potential role of wildlife in the USA in the event of a foot-and-mouth disease virus incursion. Vet Rec. (2019) 184:741. doi: 10.1136/vr.104895, PMID: 31023873

[39] Miller RS Sweeney SJ Slootmaker C Grear DA Di Salvo PA Kiser D. Cross-species transmission potential between wild pigs, livestock, poultry, wildlife, and humans: implications for disease risk management in North America. Sci Rep. (2017) 7:7821. doi: 10.1038/s41598-017-07336-z, PMID: 28798293 PMC5552697

[40] Walz E Evanson J Sampedro F VanderWaal K Goldsmith T. Planning “plan B”: the case of moving cattle from an infected feedlot premises during a hypothetical widespread FMD outbreak in the United States. Front Vet Sci. (2019) 6:484. doi: 10.3389/fvets.2019.00484, PMID: 31998764 PMC6964524

[41] Walz E Middleton J Sampedro F VanderWaal K Malladi S Goldsmith T. Modeling the transmission of foot and mouth disease to inform transportation of infected carcasses to a disposal site during an outbreak event. Front. Vet. Sci. (2020) 6:501. doi: 10.3389/fvets.2019.00501, PMID: 31993448 PMC6971117

[42] Hayer SS Ranjan R Biswal JK Subramaniam S Mohapatra JK Sharma GK. Quantitative characteristics of the foot-and-mouth disease carrier state under natural conditions in India. Transbound Emerg Dis. (2018) 65:253–60. doi: 10.1111/tbed.12627, PMID: 28251837

[43] Stenfeldt C Arzt J. The carrier conundrum; a review of recent advances and persistent gaps regarding the carrier state of foot-and-mouth disease virus. Pathogens. (2020) 9:167. doi: 10.3390/pathogens9030167, PMID: 32121072 PMC7157498

[44] Stenfeldt C Eschbaumer M Rekant SI Pacheco JM Smoliga GR Hartwig EJ. The foot-and-mouth disease carrier state divergence in cattle. J Virol. (2016) 90:6344–64. doi: 10.1128/JVI.00388-16, PMID: 27147736 PMC4936139

