# Supplementary material for: Assessment of a reconfiguration of the InterSpread Plus US national FMD model as a potential tool to analyze a foot-and-mouth disease outbreak on a single large cattle feedlot in the United States
Source: Front Vet Sci. 2023 Aug 16;10:1205485. doi: 10.3389/fvets.2023.1205485 (PMC10468568; doi:10.3389/fvets.2023.1205485)
Supplement: Supplementary file 1 [file Table_1.DOCX]

## Appendix A

Response Cost Budget

A budget for feedlot disease response cost estimation was developed by Oklahoma State University for USDA-APHIS under cooperative agreement. The 4 spreadsheet budgets provided estimated single-location response cost for the following animal types:

- Cattle
  - Cow-calf
  - Stocker
  - Feedlot
  - Dairy
- Swine
  - Commercial
  - Transitional or Small Enterprise
- Small Ruminant
  - Sheep
  - Goat
- Bison

Because of the diversity in the types of operations, multiple approaches to depopulation and disposal could be selected for an individual operation. The user has the option to select the operation type and enter the number of head for the operation. The final budget will return both a per head cost and a cost for the total operation. In addition, the user may adjust the time required in minutes per head for each response step: euthanasia, disposal, cleaning and disinfection, and vaccination.

Labor requirements and cost for each activity were based on the minutes per head entered at the top of the calculator rather than tying them directly to the method. This allows the user to account for variations in the *location* of the herd in addition to herd characteristics that would affect the time it takes to achieve each step of disease eradication on a farm.

For both euthanasia and disposal, a table with supply sheets is included. This table is used to toggle certain supplies on and off. For example, if landfill disposal is selected but lime is not needed to cover carcasses, then the “lime” supply can be toggled off by changing the value under “landfill” from “1” to “0”. Similarly, additional methods can be added using the supply sheets. This would require a few additional steps in adjusting some of the drop-down menu options but is easily accomplished.

*Euthanasia*

There are multiple euthanasia/depopulation methods that could be used on a livestock operation. The methods chosen for the calculator are all approved euthanasia methods wherein the animal is unconscious before the heart is stopped. Other methods of depopulation that may have been used in other countries, such as the use of firearms or exsanguination, were not included. The three methods in the calculator now are:

- Captive bolt to achieve unconsciousness followed by a euthanasia solution administered by syringe.
- Sedative (Xylazine) administered by syringe follow by euthanasia solution administered by syringe.
- Sedative administered by dart rifle followed by euthanasia solution administered by syringe.

For each option, supplies and equipment are specified. The pricing for the supplies and equipment are based on commercial costs from veterinary supply companies. Although some of the equipment and supplies are likely already in the National Veterinary Stockpile, any NVS supplies would need to be replaced after the event. Therefore, the cost of all supplies required was included in each cost estimate. General categories of supplies would include:

- Personal protective equipment (PPE) for all personnel involved in euthanasia
- Syringes and needles. Because of their application for euthanasia, needles are assumed to be used multiple times. For each, we included standard replacement rates for disposable syringes and needles. Two syringes (a 60ml and a 30 ml) were assumed to be used per animal to administer euthanasia solution. This is with the assumption that both speed and animal welfare will be priorities, and that two people can be moving down the line administering the solution. When a sedative is included, additional syringes and needles are added to the requirements.
- Captive bolt gun and cartridges. A 0.25 caliber captive bolt gun and bolts were included in the costs when captive bolt was used to achieve unconsciousness. The captive bolt gun cost per head was estimated using a standard depreciation method for what would normally be about a 5-year period, with a 20% depreciation in value per year. (This method was also used for other expensive equipment.) We assumed 1.3 bolts per animal since the bolt has to be placed exactly right to achieve unconsciousness, and in some animals a second bolt is necessary. This assumption may vary based on the expertise of the person administering the bolt.
- Tranquilizer gun. In some situations, having animals in a chute or alley to administer the euthanasia solution may not be ideal or even possible. An alternative method was included for using a tranquilizer dart to knock an animal unconscious before administering the euthanasia solution. Tranquilizer guns were depreciated using the same method as captive bolt guns to distribute the cost. In addition to the depreciation cost per use, other supplies included replacement syringes and rubber plungers, as well as propellant, needles, and charges.

Equipment required varied by operation type. Dairies were assumed to need little equipment aside from a truck and trailer to move personnel and supplies to the location. However, cow-calf operations were assumed to have animals in pastures. Rather than move animals out of pastures, supplies to build portable, temporary handling facilities were included in the euthanasia cost. This included a portable squeeze chute (depreciated to a cost per animal), portable cattle panels and the trucks/trailers to bring in and take out the equipment.

*Disposal*

There were multiple disposal methods that could be used on livestock operations: on-site burial, composting, landfill, and rendering. The method chosen may vary based on the size and location of the herd or on the resources already being expended at another operation. As with euthanasia, the supplies and equipment required vary based on the type of disposal method and the user entered expected time for disposal in minutes per head. Time is important for labor requirements and costs—it may take longer to create a burial pit or a compost pile in certain environments even if it requires the same amount of equipment or supplies. The pricing of supplies and equipment is based on a variety of sources but is still based on the commercial cost of either purchasing new supplies or replacing supplies used out of NVS. General categories of supplies would be:

- PPE is included for all personnel handling carcasses.
- Equipment is needed to move carcasses under all disposal methods, including chains of varying sizes and a tractor or track hoe. The tractor cost per operation included the operator and fuel and is based on a rental cost per hour tied to the expected time to complete disposal.
- Equipment for off-site disposal options (landfill and rendering) also includes a lined dump truck. Other similar methods of moving carcasses could be used (e.g. lined roll off dumpster) but this a dump truck was used as a baseline.
- Other equipment includes the trucks and trailers to move people, supplies and equipment on or off of the site.
- Plastic liners that would cover 2,000 square feet are included for multiple methods—they are needed to line trucks for moving carcasses, to line burial pits for on-site burial and for landfill burial. Lime is included to cover carcasses with the exception of composting and rendering.
- Wood chips, straw and sawdust are the carbon source available for composting in the calculator based on commercial costs as of 2019, but the cost can be quickly updated to reflect alternative carbon sources or changes in carbon supply costs.

*Cleaning and Disinfection*

Unlike both euthanasia and disposal, which was mainly variable costs per head, cleaning and disinfection (C&D) can be subject to economies of scale. This is because C&D is more closely tied to the operation facilities. For example, a commercial swine operation has extensive indoor facilities filled with complex equipment. It is expensive to C&D a such a facility as compared to a pole barn where hay is stored for a cow-calf operation. Costs for indoor facilities include hand equipment (e.g., shovels and brooms) as well as larger equipment (e.g., skid steer) to move bedding and heavy items like panels and chutes. The C&D costs are very sensitive to the time requirement.

Further, some facilities, like a pole barn, may not make sense to C&D at all. Rather, it might make more sense to simply fallow that area for a time, particularly if the weather is hot and sunny. This is particularly true of pens, windbreaks, and barns that are largely made of wood. The risk of environmental contamination by a virus may vary by the type of disease in question as well.

In all cases, the supplies to decontaminate equipment is included. The decontamination cost for all activities is currently included in the cleaning and disinfection cost. This would include a pressure washer, and the cost of vircon mix. Hand sprayers are also included for people to decontaminate after each activity.

*Vaccination*

Vaccination cost can be broken out separately from the other costs as a standalone for an un-infected farm, as opposed to the previous activities for infected farms. Class C PPE is included for all animal handlers. Vaccination supplies include a refrigerated trailer to assure cold chain, syringes and needles in addition to the vaccine dose. Syringes are multi-use, but, unlike euthanasia, needles are assumed to be single use. Containers to appropriately dispose of all needles, syringes and other supplies is included.

In addition to the administration of the vaccine, the cost of RFID tagging, and tracking cattle is included in the vaccination cost. This includes the ear tags, ear tag applicator, and RFID reader (depreciated cost).

Equipment varied by operation type. Portable pens and chutes were included in the cost for cow-calf and stocker cattle operations, but not for feedlots or dairies. Trucks and trailers were included in the equipment cost. Personnel costs were based on the minutes per head for vaccination and should include set up and break down time in the average.

*Quarantine and Surveillance*

As with vaccination, quarantine costs can stand alone for an operation. At this time, they are simply the cost for personnel and supplies to inspect herds, take samples, and meet with owners. It includes PPE and a per diem per mile that includes transportation and labor.
