# Supplementary material for: Assessment of a reconfiguration of the InterSpread Plus US national FMD model as a potential tool to analyze a foot-and-mouth disease outbreak on a single large cattle feedlot in the United States
Source: Front Vet Sci. 2023 Aug 16;10:1205485. doi: 10.3389/fvets.2023.1205485 (PMC10468568; doi:10.3389/fvets.2023.1205485)
Supplement: Supplementary file 2 [file Data_Sheet_1.pdf]

Supplementary Material

Assessment of a Reconfiguration of the Interspread Plus US National FMD Model as a Potential Tool to Analyze a Foot-and-Mouth Disease Outbreak on a Single Large Cattle Feedlot in the United States

Sarah R. Mielke*[a], Rigney, Columb[a], Hagerman, Amy D.[b], Boyer, Timothy C.[a], Delgado, Amy H. [a], Arzt, Jonathan[c], Holmstrom, Lindsey K.[d]

*Corresponding author
sarah.mielke@usda.gov

1 Supplementary Tables:

Table 1: Prevalence estimates of Bovine Respiratory Disease for cattle on feedlots drawn from the NAHMS 2011 Part 4 report.

Disease Reference			
**Bovine Respiratory Disease (BRD) (shipping fever)**			
**Cattle Group**	**Prevalence (%)**	**Treated (%)**	**Data Source**
**Calves**	21.2	18.9 (89.6% of Prevalence)	NAHMS 2011 Part 4, feedlot report on cattle affected and cattle treated for BRD (USDA, 2011)
**Yearlings**	8.8	7.4 (84.1% of Prevalence)	
**Cows/Bulls**	8.8	7.4 (84.1% of Prevalence)	

Table 2: Calculation and source to estimate turnover rates in the simulated feedlot.

Animal type	Total pens	Days on Feed (DoF)	Yearly turnover rate	Resource
**Calf fed**	246	214	$\frac{365}{214} = 1.70$	Beef Cattle Institute (BCI) data regarding cattle movement onto and off feedlots and weight gain.
**Yearling**	238	129	$\frac{365}{129} = 2.83$	
**Cow/Bull**	11	60	$\frac{365}{60} = 6.08$	

Table 3: Estimated number of movements from and to a treatment location on our simulated feedlot based on data from BCI.

Animal Type	Total Number of Animals	Yearly Turnover rate	Year Total Number of Animals	# Daily Movements	Percent of cattle moved, treated for BRD (%)	Daily Treatment Movements	Number of Movements to/from possible treatment pens
**Calf fed**	24600	1.70	41958	115	18.9	22	$\frac{22}{9} = 2.44$
**Yearling**	24400	2.83	69038	189	7.4	14	$\frac{14}{9} = 1.55$
**Cow/Bull**	1000	6.08	6083	17	7.4	1	$\frac{1}{9} = 0.11$

Table 4: Model specifications and assumptions for control measures implemented in the model scenarios.

Scenario	Control	Model Section	Value	Notes
**All**	72-hour standstill	Movement Restriction 1	0.5; Undetected	Probability movement is restricted for undetected pens including to feedlot, receiving pens to feeding, and between hospital and feeding pens.
	72-hour standstill	Movement Restriction 2	0; Detected	Probability movement is restricted for indirect contacts between detected pens (indirect pen-riders and veterinarians) Feed trucks are not restricted
	Post 72h standstill	Movement Restriction 3	0.9; detected or undetected	Probability movement is restricted for all feedlot pens excluding shipping pens (these are in a separate movement restriction).
	Post 72h standstill	Movement Restriction 4	0; detected	Probability movement is restricted for indirect movements, these movements are an essential function and are therefore not restricted at any time – the ability to alter this exists within the model for enhanced biosecurity planning.
	72-hour standstill	Movement Restriction 5	0.8; detected	Probability movement is restricted for detected pens including to feedlot, receiving to feeding pens, and between hospital and feeding pens.
	Standstill	Movement Restriction 6	1.0; detected or undetected	Probability movement is restricted for detected and undetected pens including movement to shipping pens and off feedlot to slaughter plants.
**Stamp-out, Firebreak**	Depopulation	Resource Depopulation	12, 10, 7	The number of pens depopulated per time period. Time periods (tp) include: tp 1-2, tp 3-29, and tp 30-365, respectively.
**Firebreak, Harvest (w/ vaccine)**	Vaccination	Resource Vaccination	0, 5, 8, 5, 3, 0, 13	The number of pens vaccinated per time period. There are 7 values used across the time period 1 – 365 to reflect fluctuations in vaccine resource and capabilities on the feedlot.
**Harvest w/ and w/o vaccine)**	Send to slaughter	Resource Depopulation	4	The number of pens sent to slaughter per time period over the course of the simulation (1-365 tp).
		Time Period Trigger with Set State	30	The number of days post detection that a pen can be set as selected for harvest.

Assumptions: Movement restriction for a feedlot maintains a 72-hour standstill separate from a full standstill to account for the potential for cattle shipments returning to the feedlot and movement around the feedlot for initial response activities (i.e., isolation of pens or cattle). However, movements off the feedlot are completely restricted, due to the expectation that when the feedlot has any detections these movements would cease.

Table 5: The low-cost scenario implementing captive bolt with euthanasia solution for depopulation and burial on-site for disposal illustrates that the two harvest scenarios result in the lowest overall cost related to response activities (indicated in green).

Low-Cost Option

**Scenario**	Completion Time (min/head(hd)*	Depopulation	Firebreak	Firebreak-NV	Harvest	Harvest-NV
**Total Cattle Depopulated**	---	54790	47490	54790	0	0
**Total Cattle Vaccinated**	---	0	11060	0	54790	0
**Total Cattle Harvested**	---	0	7300	0	54790	54790
**Euthanasia Method**	---	Captive bolt w/ Euthanasia Solution	Captive bolt w/ Euthanasia Solution	Captive bolt w/ Euthanasia Solution	Captive bolt w/ Euthanasia Solution	Captive bolt w/ Euthanasia Solution
**Disposal Method**	---	on-site burial	on-site burial	on-site burial	on-site burial	on-site burial
**Euthanasia Cost**	3 fire; 2 others	2,532,987.57	2,332,959.86	2,532,987.57	0.00	0.00
**Disposal Cost**	2	1,097,887.68	951,610.44	1,097,887.68	0.00	0.00
**Cleaning and Disinfecting Cost**	2	177,858.18	154,292.69	177,858.18	177,858.18	177,858.18
**Vaccination Cost**	3	0.00	133,773.17	0.00	662,511.84	0.00
Indemnity ($1355/hd)	---	*74,240,450.00*	*64,348,950.00*	*74,240,450.00*	*0.00*	*0.00*
**Lowest Week Market Value (2020)^ ($1178.87/hd)**	---	**0.00**	**8,605,751.00**	**0.00**	**64,590,287.30**	**64,590,287.30**
Cost w/o Indemnity or market value	---	3,808,733.43	3,572,636.16	3,808,733.43	840,370.02	177,858.18
**Total Cost**	---	78,049,183.43	76,527,337.16	78,049,183.43	65,430,657.32	64,768,145.48

*Completion Time is minutes/head used in our cost calculator to complete activities. This timing is based on the conversion of national FMD model parameters to a single feedlot

^ Using the lowest week from 2020 for a market value on cattle we estimated the cost paid to producers for cattle held to a recovery state or end of outbreak. This accounts for the cost associated with animal production needs and the reduced value expected for recovered or vaccinated cattle.

Table 6: The high-cost scenario implementing dart gun tranquilizer with euthanasia solution for depopulation and landfill for disposal illustrates that the two harvest scenarios result in the lowest overall cost related to response activities(indicated in green).

High-Cost Option

**Scenario**	Completion Time (min/head(hd) *	Depopulation	Firebreak	Firebreak-NV	Harvest	Harvest-NV
**Total Cattle Depopulated**	---	54790	47490	54790	0	0
**Total Cattle Vaccinated**	---	0	11060	0	54790	0
**Total Cattle Harvested**	---	0	7300	0	54790	54790
**Euthanasia Method**	---	dart gun tranquilizer with euthanasia solution	dart gun tranquilizer with euthanasia solution	dart gun tranquilizer with euthanasia solution	dart gun tranquilizer with euthanasia solution	dart gun tranquilizer with euthanasia solution
**Disposal Method**	---	landfill	landfill	landfill	landfill	landfill
**Euthanasia Cost**	3 fire; 2 others	2,864,490.19	2,614,173.47	2,864,490.19	0.00	0.00
**Disposal Cost**	2	7,761,438.46	6,727,388.43	7,761,438.46	0.00	0.00
**Cleaning and Disinfecting Cost**	2	177,858.18	154,292.69	177,858.18	177,858.18	177,858.18
**Vaccination Cost**	3	0.00	133,773.17	0.00	662,511.84	0.00
**Indemnity ($1355/hd)**	---	74,240,450.00	64,348,950.00	74,240,450.00	0.00	0.00
**Lowest Week Market Value (2020)^ ($1178.87/hd)**	---	**0.00**	**8,605,751.00**	**0.00**	**64,590,287.30**	**64,590,287.30**
**Cost w/o Indemnity or market value**	---	10,803,786.83	9,629,627.76	10,803,786.83	840,370.02	177,858.18
**Total Cost**	---	85,044,236.83	82,584,328.76	85,044,236.83	65,430,657.32	64,768,145.48

*Completion Time is minutes/head used in our cost calculator to complete activities. This timing is based on the conversion of national FMD model parameters to a single feedlot

^ Using the lowest week from 2020 for a market value on cattle we estimated the cost paid to producers for cattle held to a recovery state or end of outbreak. This accounts for the cost associated with animal production needs and the reduced value expected for recovered or vaccinated cattle.

Table 7: Convergence measures for the iteration sets 50-150 and 50 - 200 with calculation of the percent difference for the median and 90th percentile.

Scenario	Median 50-150	Median 50 - 200	% difference (Median)	90th percentile 50-150	90th percentile 50-200	% difference (90th percentile)
**Burn-through**	31	30	-3.225806	31	31	0
**Depopulation**	33	33	0	33.5	33	-1.515152
**Firebreak**	46	46	0	50	50	0
**Firebreak-NV**	34	33.5	-1.470588	34	34	0
**Harvest**	35	35	0	36	35.5	-1.408451
**Harvest-NV**	31	31.5	1.6129032	32	32	0

Table 8: The comparison of total cost between each scenario, with the scenario in column 1 as the reference scenario. Negative values indicate that column 2 scenarios cost more, while positive values indicate that column 1 scenarios had a higher total cost.

Low-Cost Comparison			High-Cost Comparison		
**Scenario 1 - Scenario 2 = Cost Difference**			**Scenario 1 - Scenario 2 = Cost Difference**		
Scenario 1	Scenario 2	Cost Difference	Scenario 1	Scenario 2	Cost Difference
depopulation	firebreak	1,521,846.27	depopulation	firebreak	2,459,908.07
depopulation	firebreak-NV	0.00	depopulation	firebreak-NV	0.00
depopulation	harvest	12,618,526.11	depopulation	harvest	19,613,579.51
depopulation	harvest-NV	13,281,037.95	depopulation	harvest-NV	20,276,091.35
firebreak	firebreak-NV	-1,521,846.27	firebreak	firebreak-NV	-2,459,908.07
firebreak	harvest	11,096,679.84	firebreak	harvest	17,153,671.44
firebreak	harvest-NV	11,759,191.68	firebreak	harvest-NV	17,816,183.28
firebreak-NV	harvest	12,618,526.11	firebreak-NV	harvest	19,613,579.51
firebreak-NV	harvest-NV	13,281,037.95	firebreak-NV	harvest-NV	20,276,091.35
harvest	harvest-NV	662,511.84	harvest	harvest-NV	662,511.84

2 Supplementary Figures

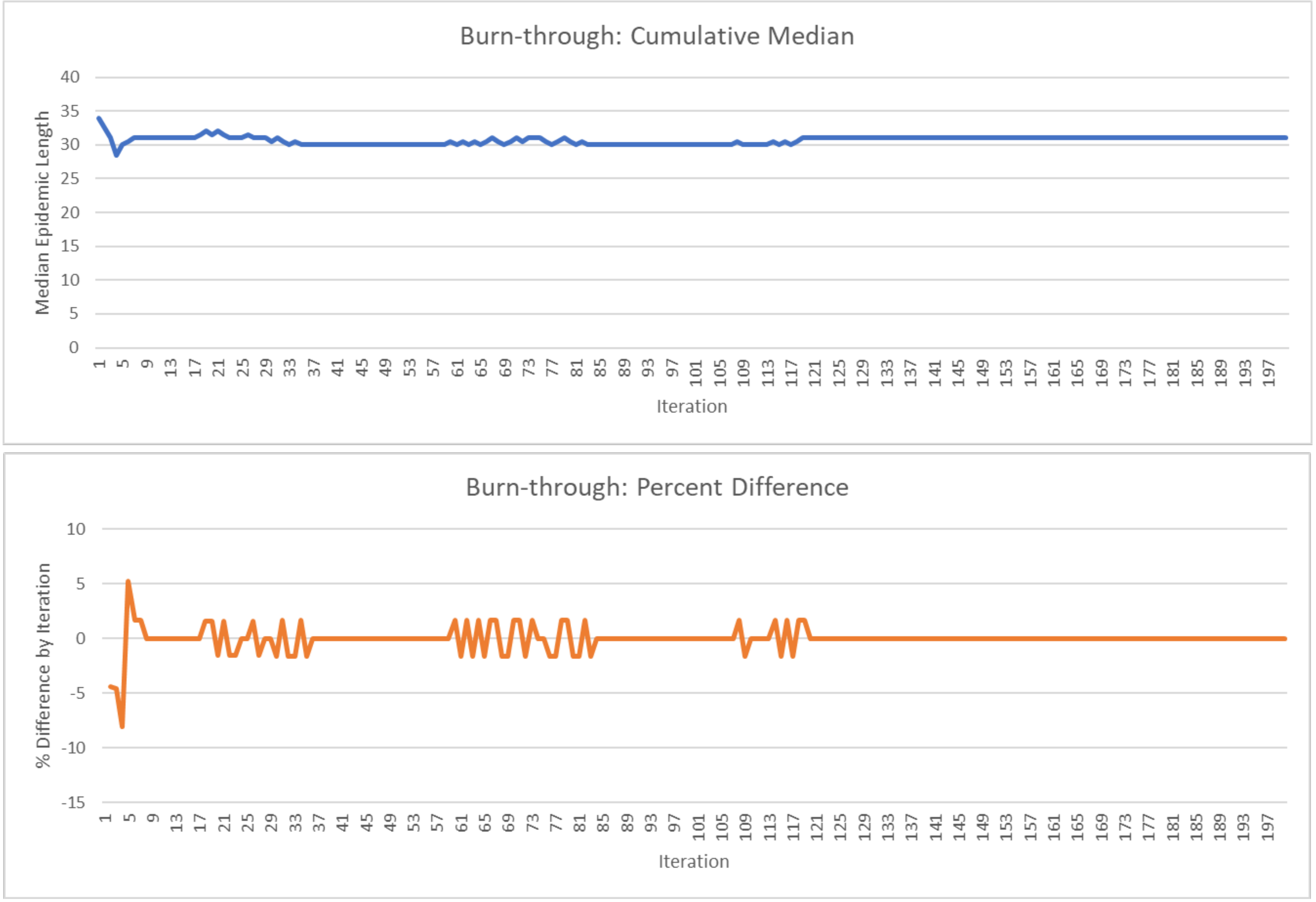

Figure S1: The cumulative median and percent difference in cumulative medians for the burn-through scenario showing the convergence with a <5% difference.

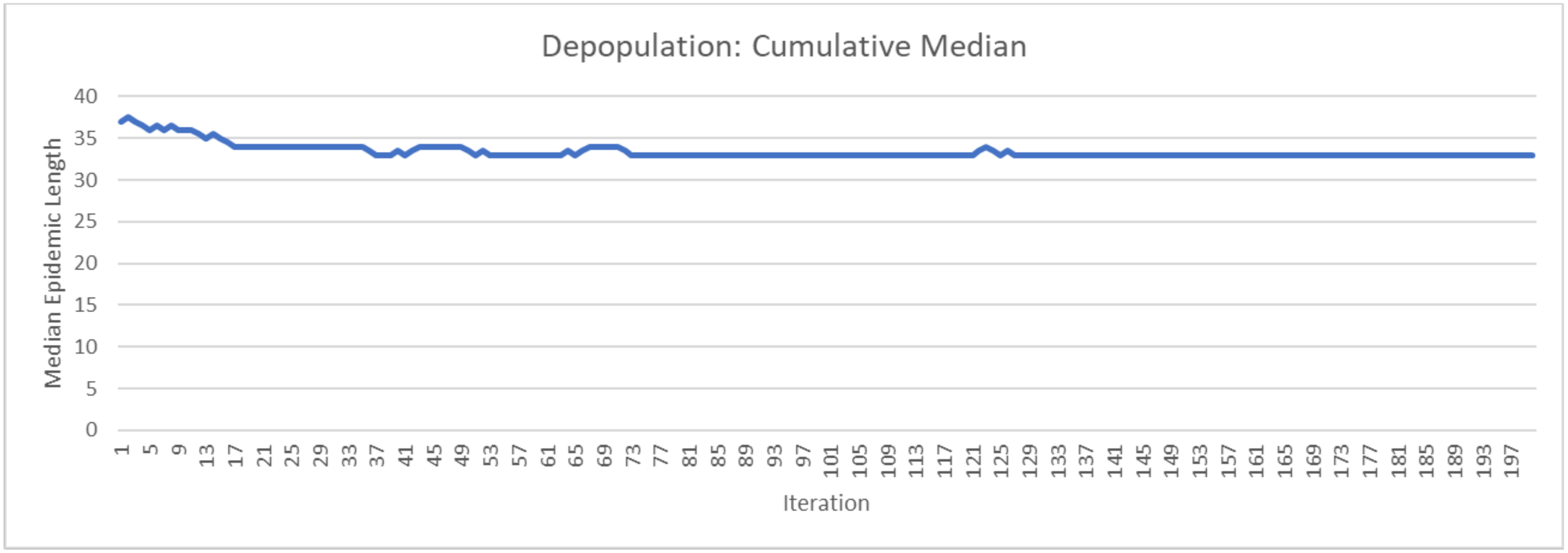

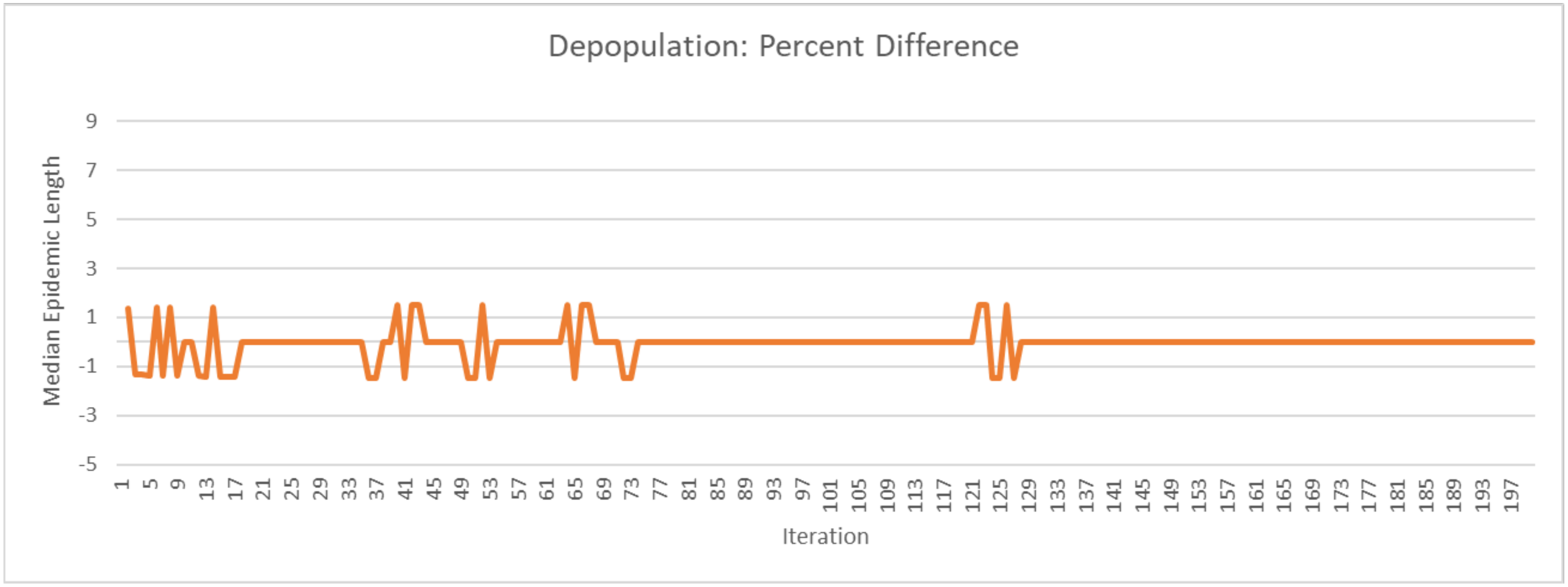

Figure S2: The cumulative median and percent differences for the cumulative medians for the depopulation scenario showing convergence with a <5% difference.

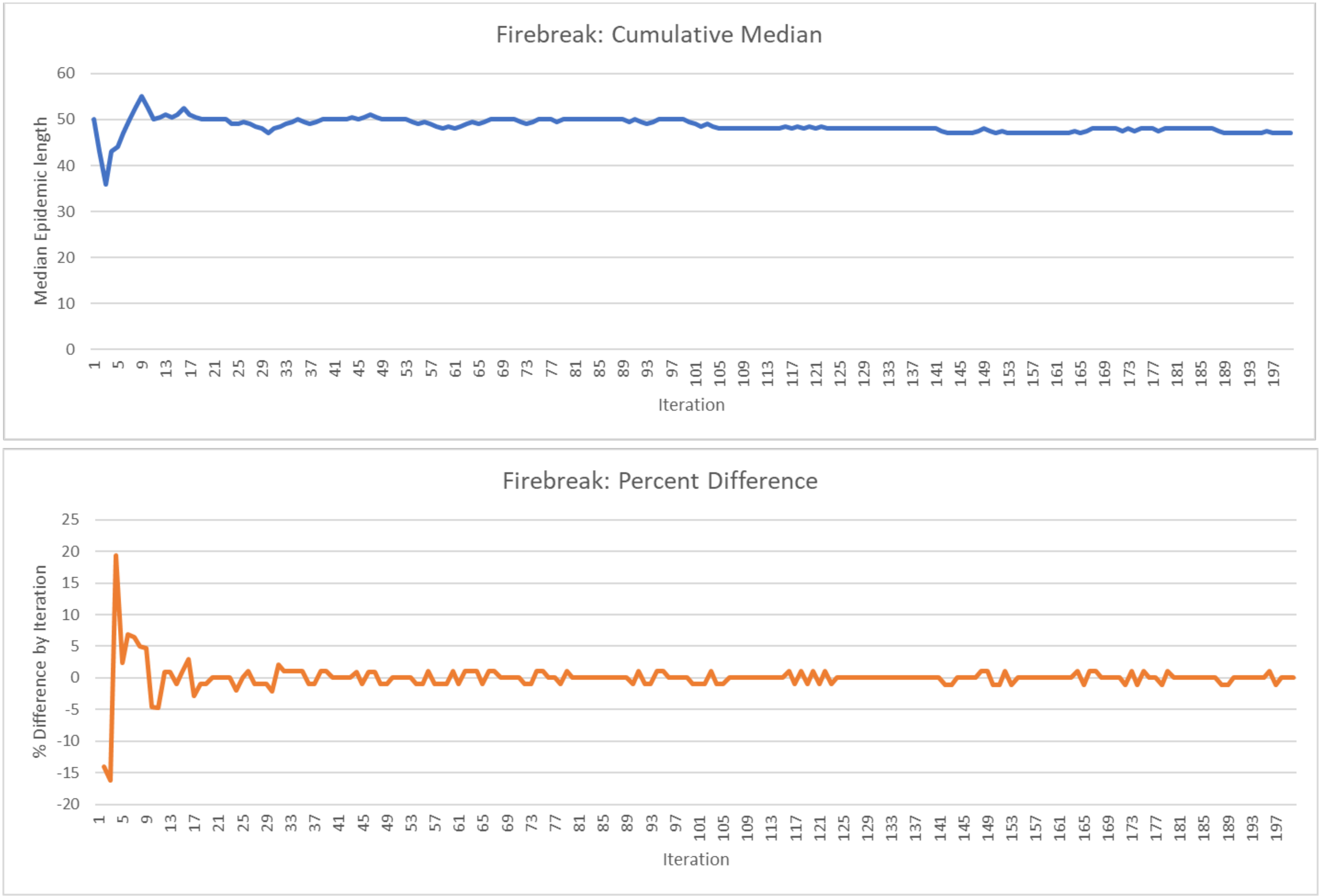

Figure S3: The cumulative median and percent differences for the cumulative medians for the firebreak scenario showing convergence with a <5% difference.

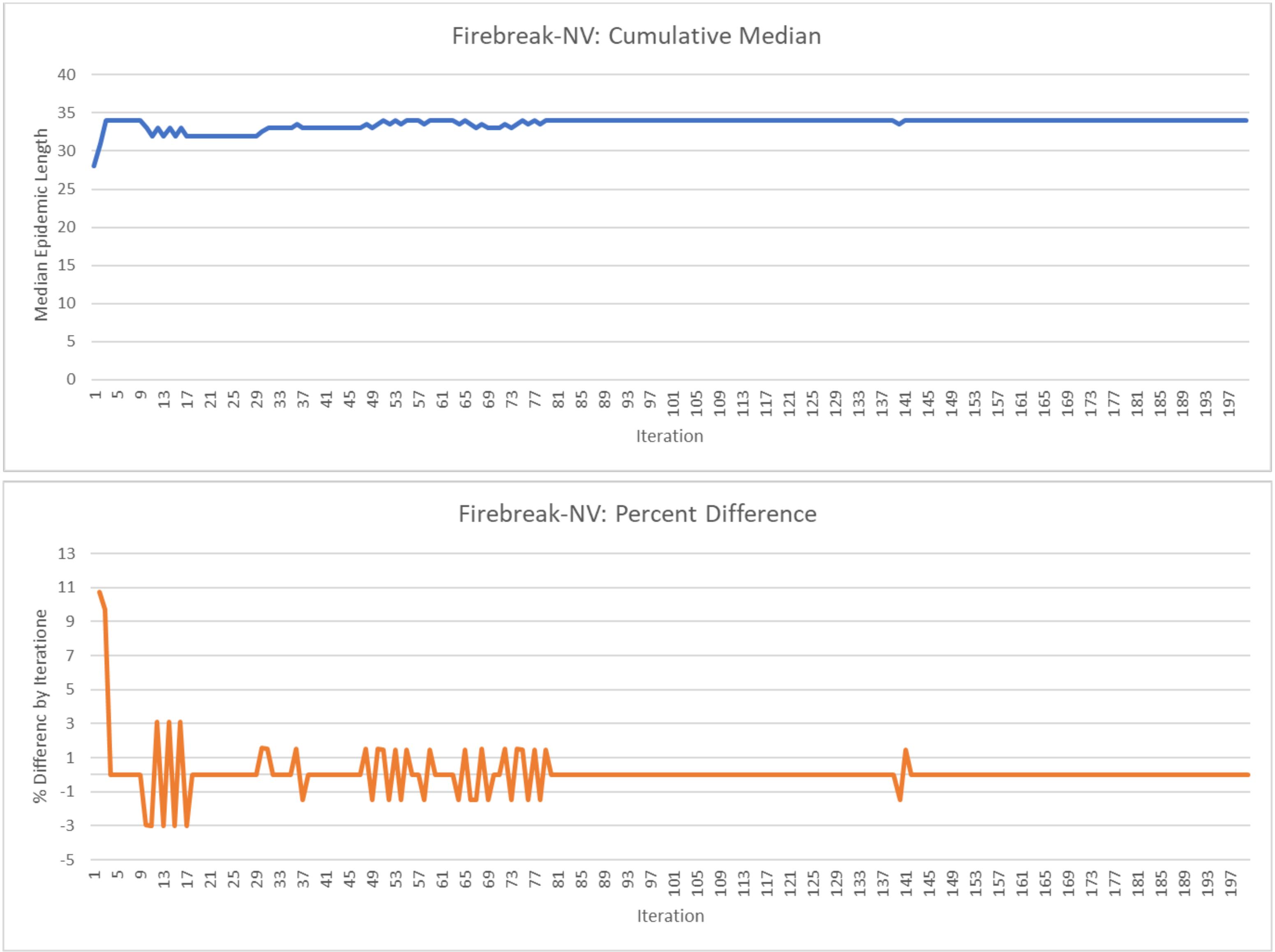

Figure S4:The cumulative median and percent differences for the cumulative medians for the firebreak-NV scenario showing convergence with a <5% difference.

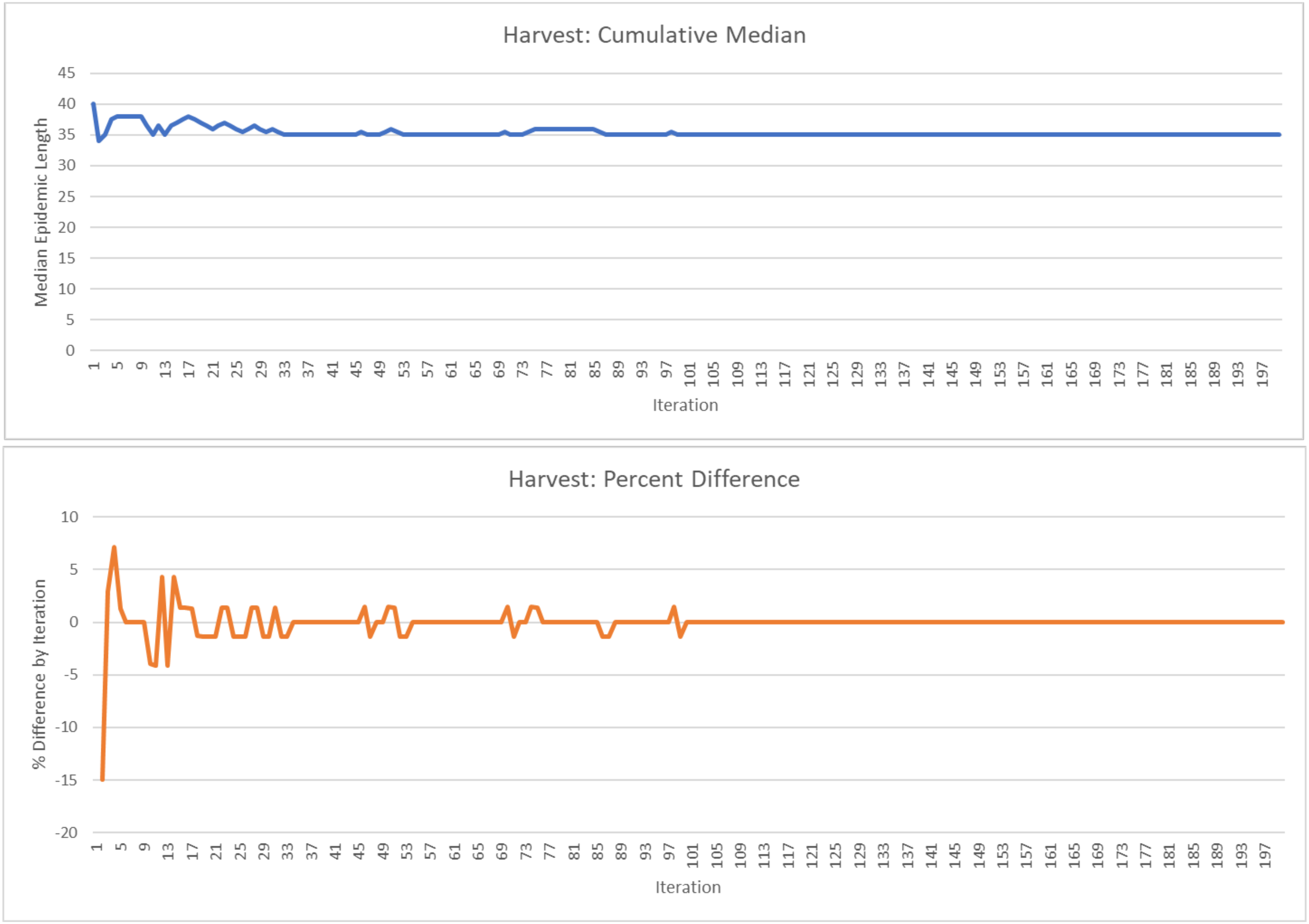

Figure S5:The cumulative median and percent differences for the cumulative medians for the harvest scenario showing convergence with a <5% difference.

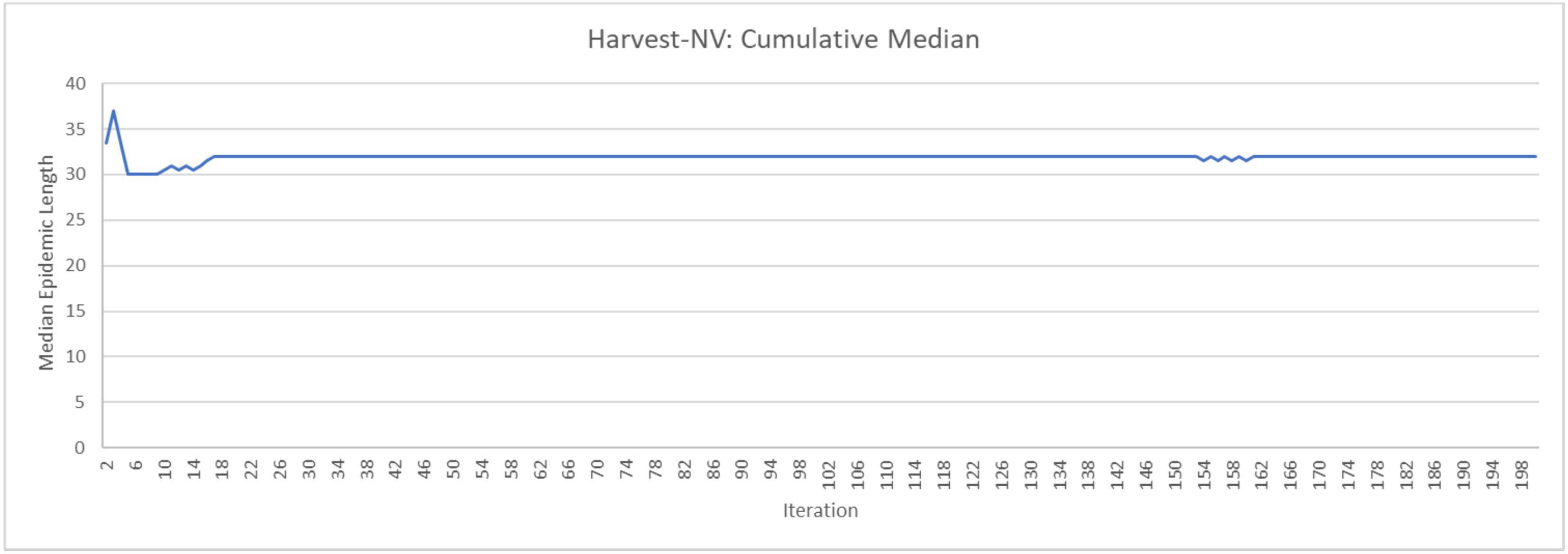

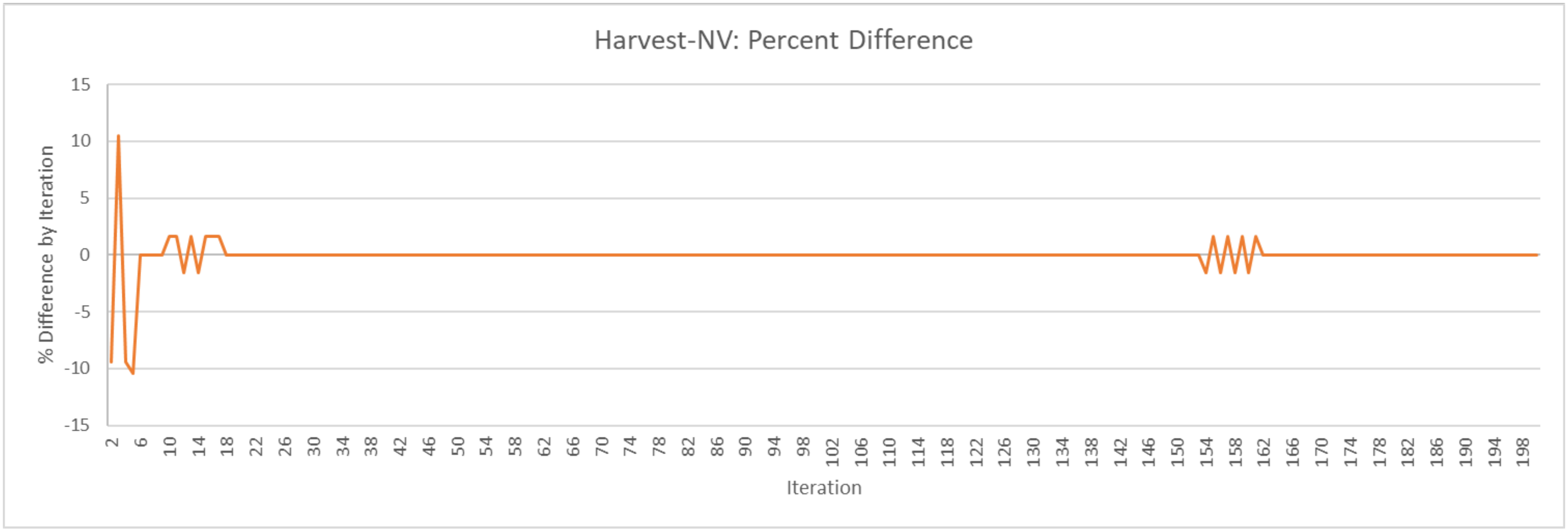

Figure S6:The cumulative median and percent differences for the cumulative medians for the harvest-NV scenario showing convergence with a <5% difference.

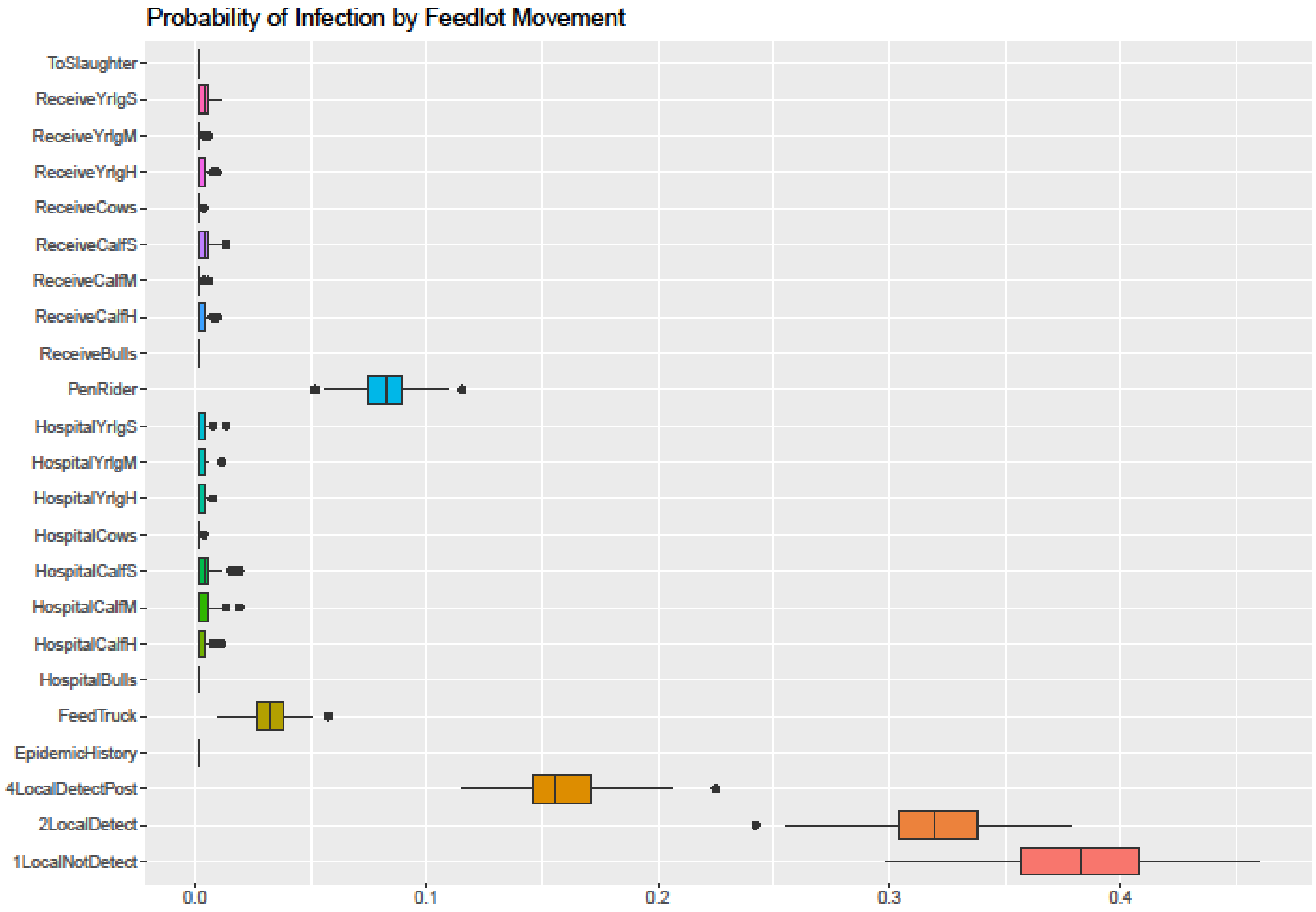

Figure 7: The proportion of infectious spread resulting from the various movements in the burn-through scenario (burn).

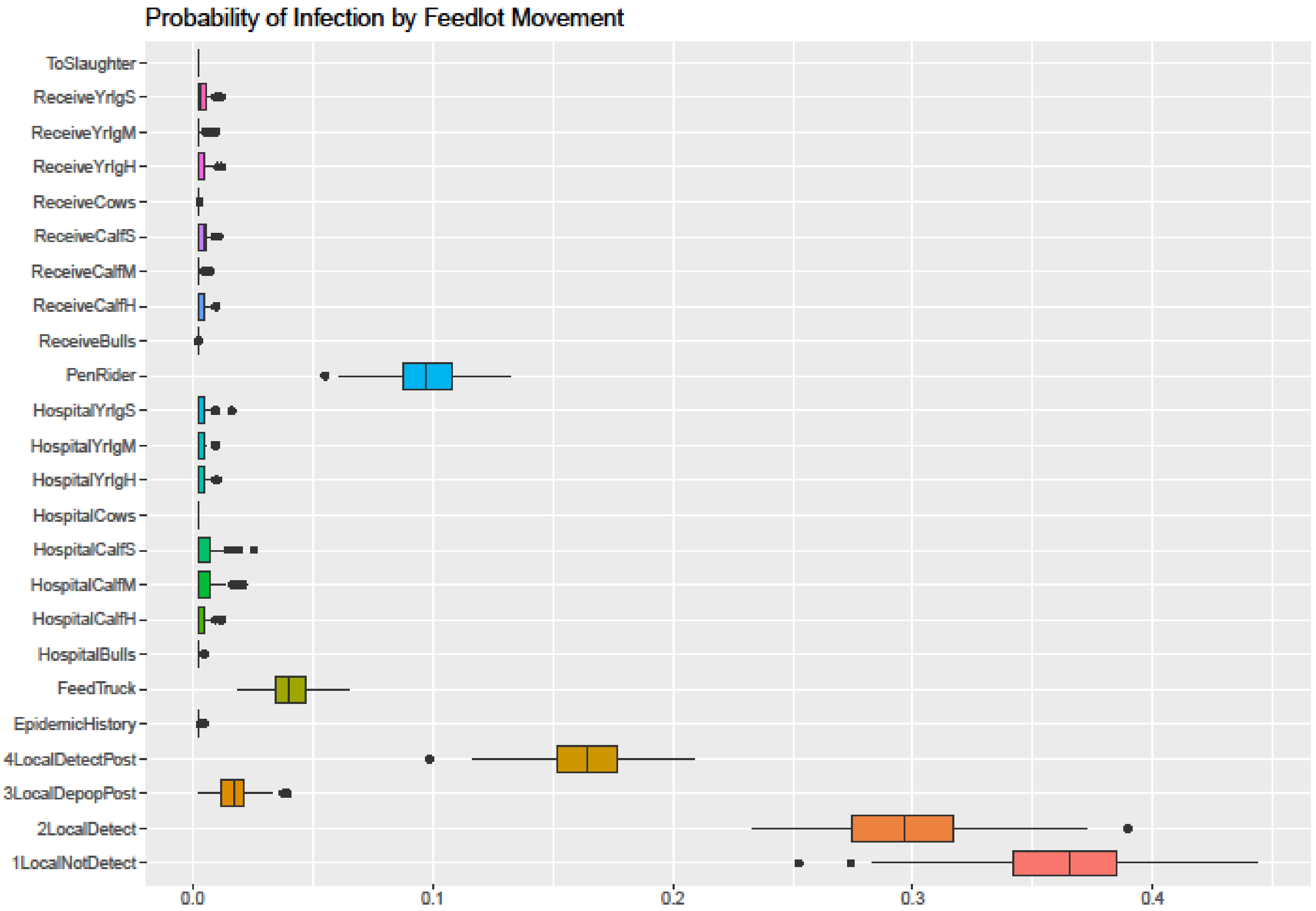

Figure 8: The proportion of infectious spread resulting from the various movements in the depopulation scenario (depopulation).

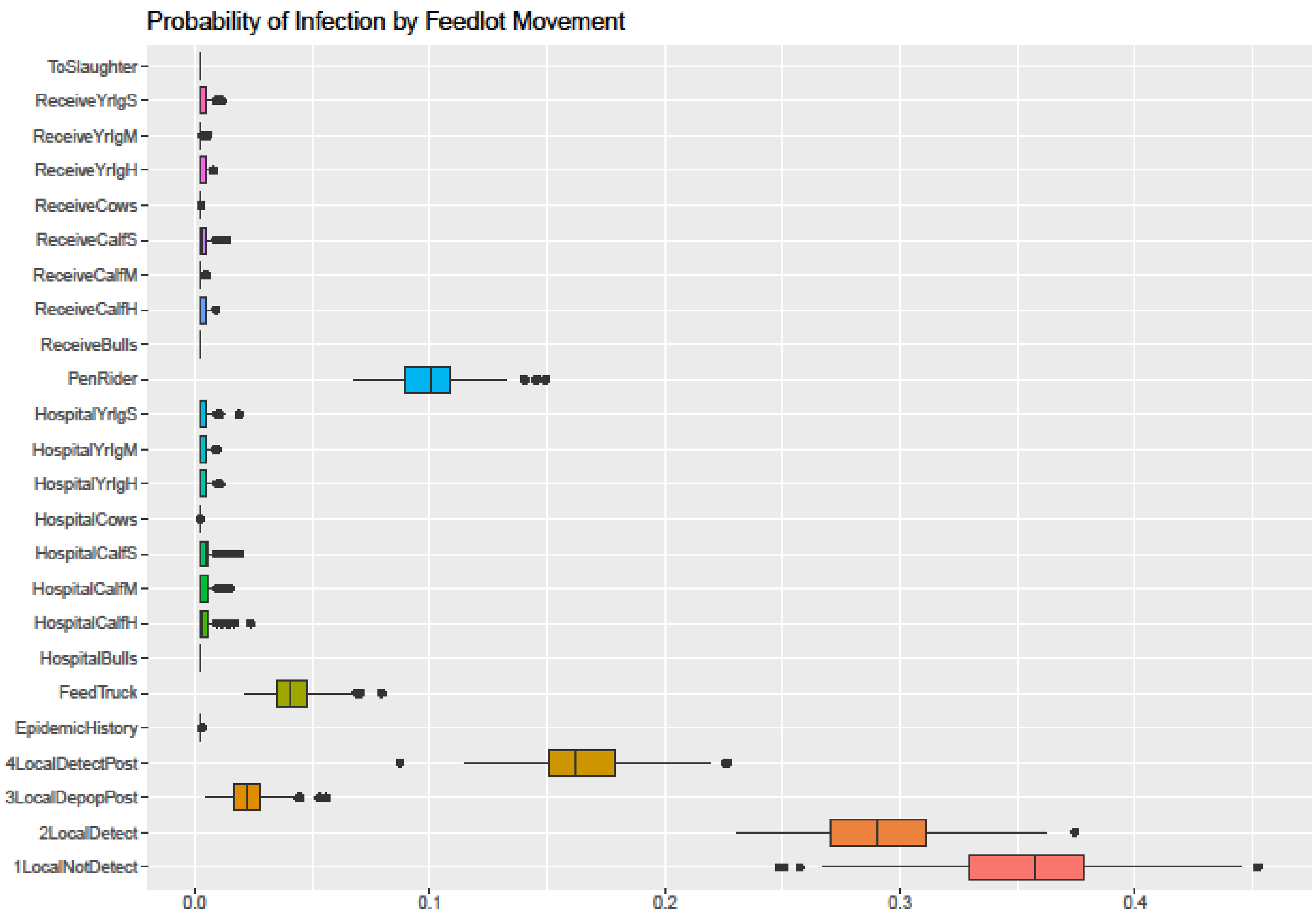

Figure 9: The proportion of infectious spread resulting from the various movements in the firebreak scenario (fire).

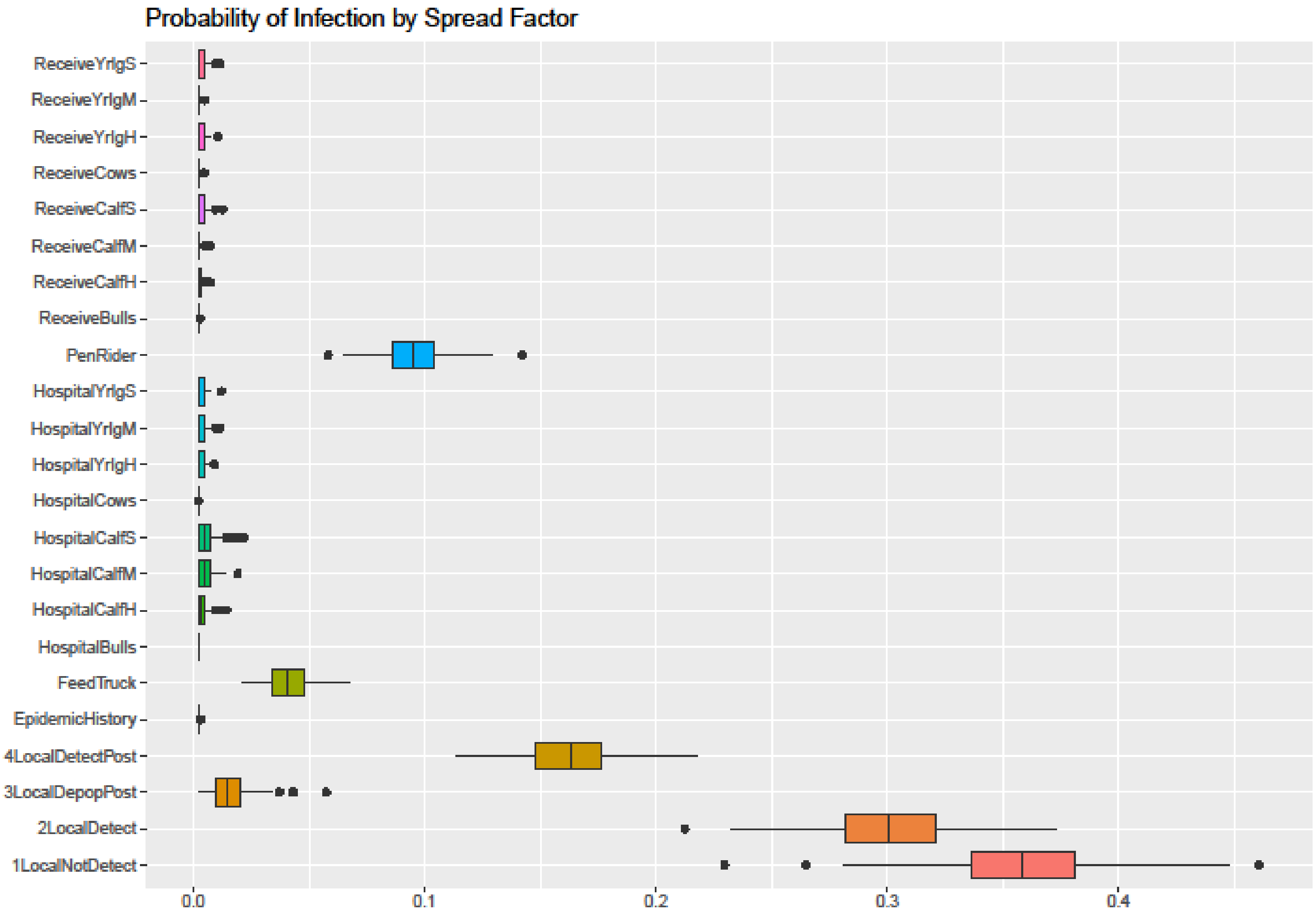

Figure 10: The proportion of infectious spread resulting from the various movements in the firebreak scenario without vaccination (fireNV).

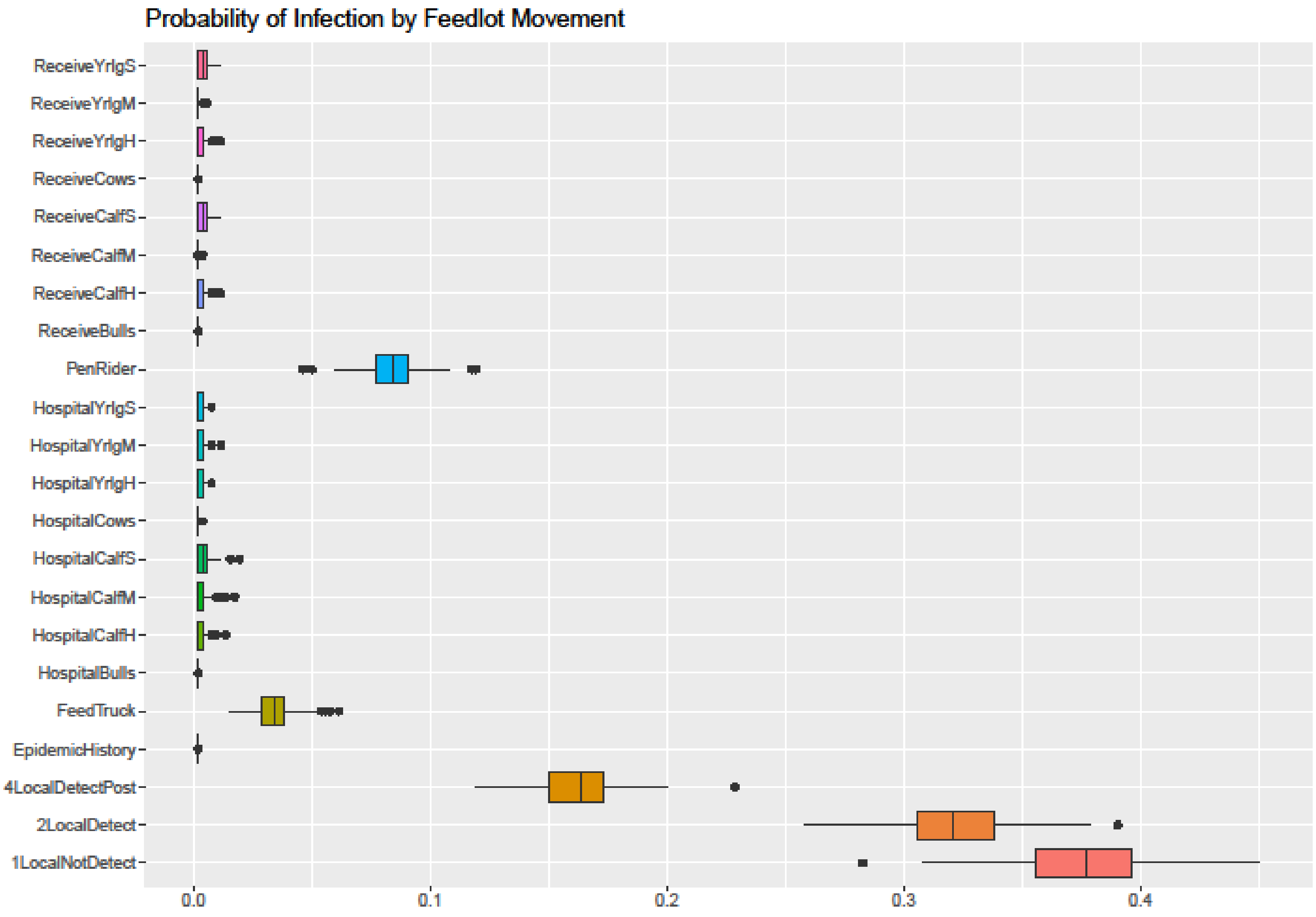

Figure 11: The proportion of infectious spread resulting from the various movements in the harvest scenario (harvest).

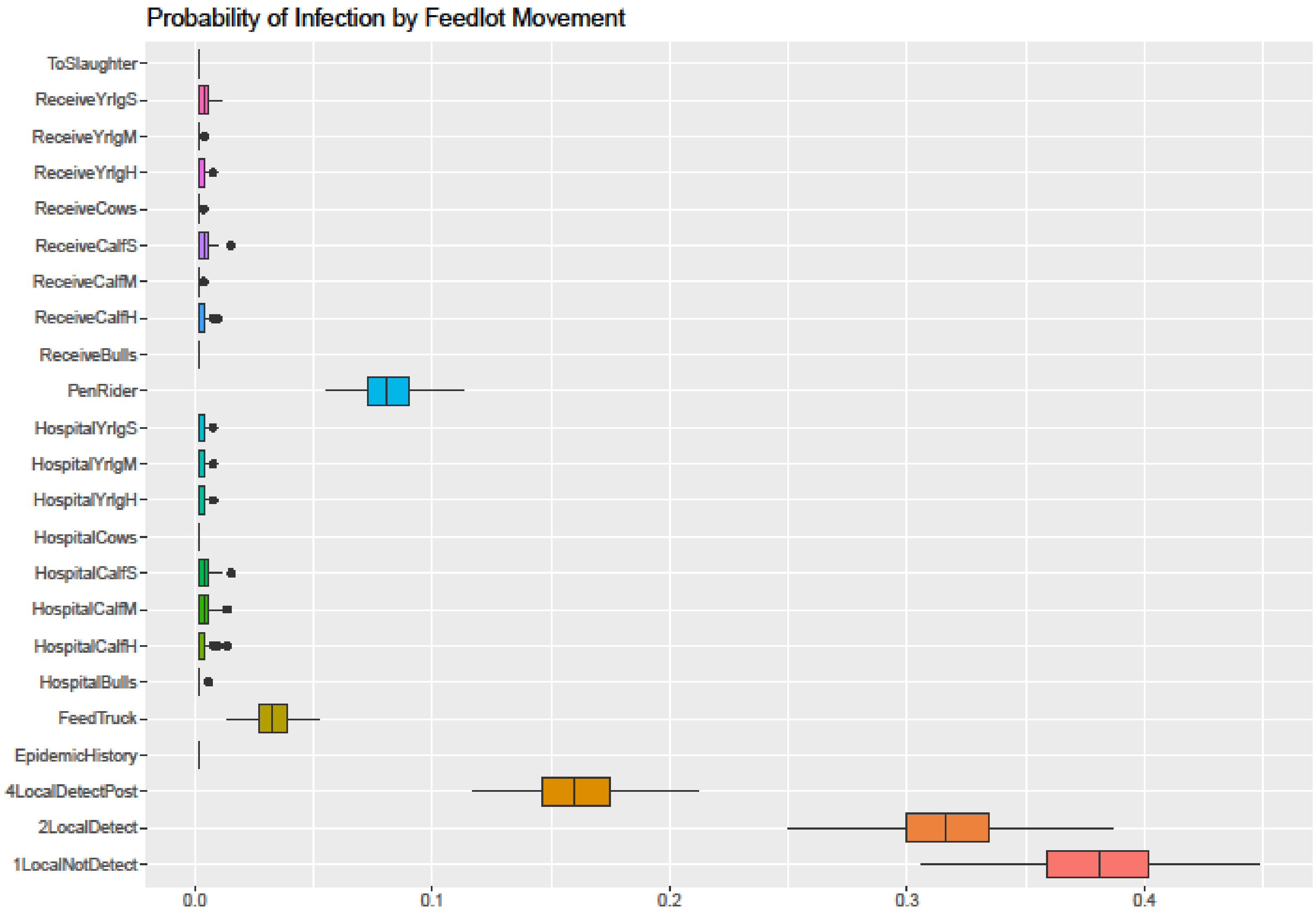

Figure 12: The proportion of infectious spread resulting from the various movements in the harvest scenario without vaccination (harvestNV).

Bibliography

USDA. (2011). *Feedlot 2011 Part IV: Health and Health Management on U.S. Feedlots with a Capacity of 1,000 or More Head*. Retrieved from Fort Collins CO:
